# A primer on the use of mouse models for identifying direct sex chromosome effects that cause sex differences in non-gonadal tissues

**DOI:** 10.1186/s13293-016-0115-5

**Published:** 2016-12-13

**Authors:** Paul S. Burgoyne, Arthur P. Arnold

**Affiliations:** 1 Stem Cell Biology and Developmental Genetics, Mill Hill Laboratory, Francis Crick Institute, The Ridgeway, London, NW7 1AA UK; 2 Department of Integrative Biology and Physiology, and Laboratory of Neuroendocrinology of the Brain Research Institute, University of California, Los Angeles, 610 Charles Young Drive South, Los Angeles, CA 90095-7239 USA

**Keywords:** Sex determination, Sexual differentiation, Sex chromosomes, X chromosome, Y chromosome, Testosterone, Estradiol, Gonadal hormones

## Abstract

In animals with heteromorphic sex chromosomes, all sex differences originate from the sex chromosomes, which are the only factors that are consistently different in male and female zygotes. In mammals, the imbalance in Y gene expression, specifically the presence vs. absence of *Sry*, initiates the differentiation of testes in males, setting up lifelong sex differences in the level of gonadal hormones, which in turn cause many sex differences in the phenotype of non-gonadal tissues. The inherent imbalance in the expression of X and Y genes, or in the epigenetic impact of X and Y chromosomes, also has the potential to contribute directly to the sexual differentiation of non-gonadal cells. Here, we review the research strategies to identify the X and Y genes or chromosomal regions that cause direct, sexually differentiating effects on non-gonadal cells. Some mouse models are useful for separating the effects of sex chromosomes from those of gonadal hormones. Once direct “sex chromosome effects” are detected in these models, further studies are required to narrow down the list of candidate X and/or Y genes and then to identify the sexually differentiating genes themselves. Logical approaches to the search for these genes are reviewed here.

**Electronic supplementary material:**

The online version of this article (doi:10.1186/s13293-016-0115-5) contains supplementary material, which is available to authorized users.

## Background

In animals with an unmatched (heteromorphic) pair of sex chromosomes, all sex differences in the phenotype originate from the unequal effects of the sex chromosomes because they are the only factors that consistently differ between male and female zygotes. In species such as mammals in which the sex chromosomes are XX and XY, a fundamental goal is to identify the sex-biasing effects of the two sex chromosomes on phenotypes. This review discusses the strategies for identifying X and Y genes or mechanisms that cause sexual bias, using mouse models that differ in their sex chromosome complement, including differences in the parental source of the X chromosome.

Primary among the sexually unbalanced effects of X and Y genes are those that cause sexual differentiation of the gonads. In mammals, the most important sex differences in the gonads are thought to be initiated by the Y-linked gene *Sry*, which is expressed in the indifferent gonadal ridge of males and induces there a cascade of molecular and cellular events that commit that tissue to a testicular fate [1–4]. In the absence of *Sry* in the XX female, ovaries develop through an active developmental program that includes inhibition of testicular pathways [5]. Therefore, the developmentally limited male-specific expression of *Sry* in the gonads sets up a lifelong sex difference in secretion of testicular vs. ovarian hormones. Because sex steroid hormones, especially androgens and estrogens, are considered the major class of proximate signals that act throughout the body to cause sexual differentiation, the *Sry* effect has long been thought to be primarily responsible for determining the sexual phenotype of most mammalian species.

In addition to the effects of *Sry* on gonadal tissues, X and Y genes may be expressed at different levels in XX and XY non-gonadal cells as a consequence of their imbalance in the genomes of all XX and XY cells. By virtue of this imbalance, X and Y genes are known to cause numerous sex differences in phenotype [6–11]. These X and Y gene effects are here called “direct” effects of the sex chromosomes, because the sex bias in the X and Y gene expressions acts directly to cause sex differences in non-gonadal tissues instead of indirectly via an action on the gonads to induce sex differences caused by gonadal hormones [12]. Historically, it has been much easier to study the effects of gonadal hormones, which can be administered or blocked or withdrawn by simple experimental procedures. It has been much more difficult to discover the direct sex-biased effects of the X and Y genes on non-gonadal tissues, because altering the sex chromosome complement usually causes changes in the type or function of the gonads, and therefore introduces confounding differences in the level of gonadal secretions.

Here, we review mouse models (see also [13]) that enable the separation of sex chromosome complement effects from the hormonal confounds, thus facilitating the identification of direct sex chromosome effects (direct SCEs) that contribute to sex differences. We focus especially on direct SCEs that are expected to contribute to sex differences in the function of normal XX and XY cells. The goal of our studies, and of this review, is not only to discuss how to detect direct SCEs but also to map out a strategy for identifying the X or Y genes that cause sex differences in cells. The strategy represents a logic tree, which progressively narrows down the potential candidate genes responsible for direct SCEs.

## Anatomy of the mouse sex chromosomes and possible causes of sex differences in phenotype

**Fig. 1:**
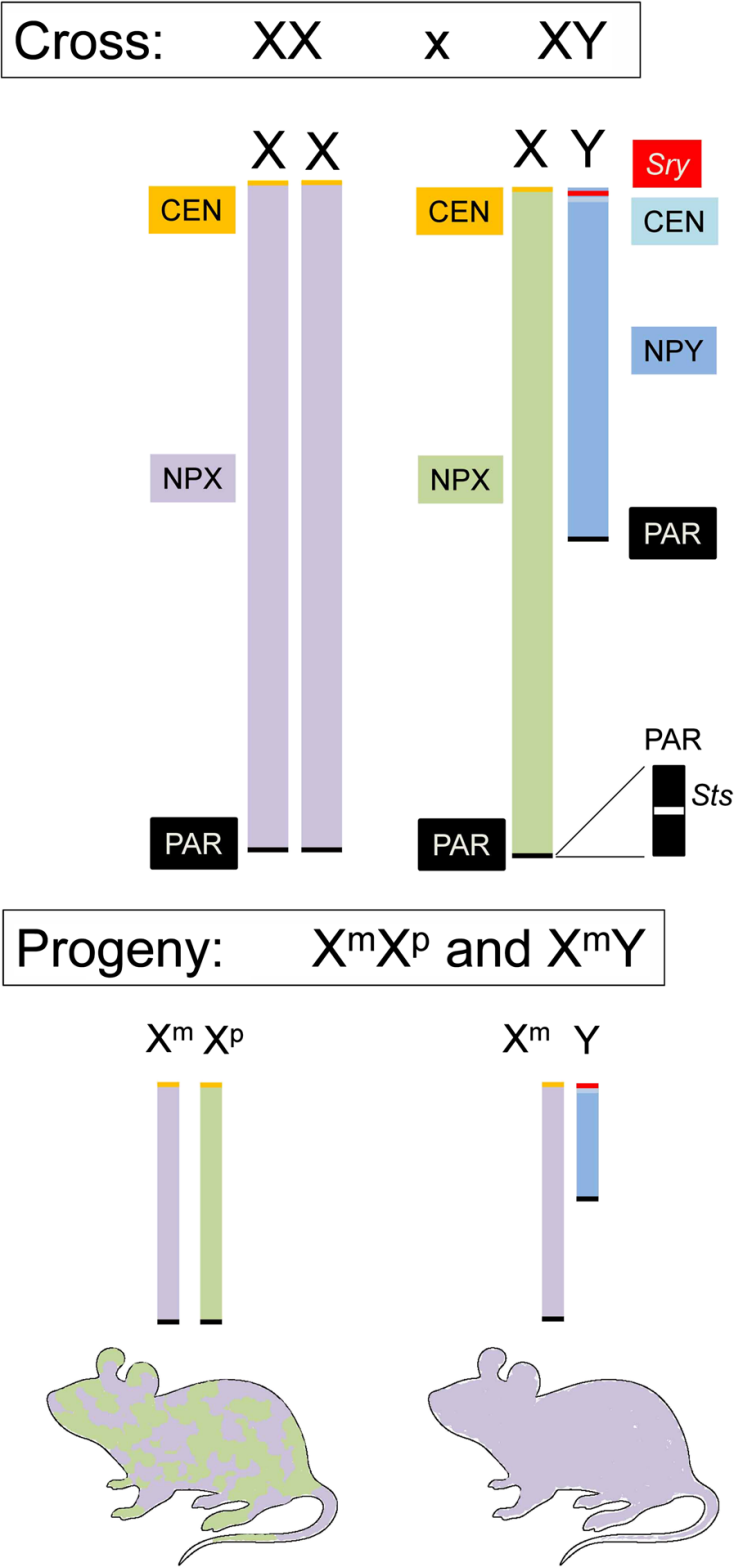
Major parts of the mouse X and Y chromosomes. *Top*: The 170-Mb X chromosome is divided into the pseudoautosomal region (PAR), containing the *Sts* gene and a few others, and the non-PAR region (NPX). The 89-Mb Y chromosome comprises the PAR and non-PAR Y (NPY). The *Sry* gene is on the Y chromosome short arm. *Bottom*: XX progeny inherit X chromosomes from the mother (X^m^) and father (X^p^). The XX mouse is a mosaic of cells expressing one of the parental X chromosomes. *Green* shows the patches of tissues in which X^p^ is active, and *lavender* shows the patches in which X^m^ is active. The XY mouse inherits the X^m^ and expresses only that X chromosome in all tissues. *CEN* centromere

The mammalian sex chromosomes are thought to have evolved from an ancestral pair of autosomes. One autosome, the proto-Y chromosome, acquired a dominant male-determining locus, which led to the loss of recombination with the proto-X, and ultimately, to the wholesale loss of gene-encoding DNA from the Y chromosome [14, 15]. However, there have been gene additions to the X and/or Y chromosome subsequent to their divergence [16–20]. The present-day Y chromosome is usually small and gene-poor relative to the X chromosome [21, 22]. However, the mouse Y has an unusually high gene count as a result of massive gene amplification that is thought to be driven by an ongoing post-meiotic X-Y genomic conflict [22, 23]. During meiosis, the X and Y chromosomes pair at the pseudoautosomal regions (PARs), thus enabling the X and Y PARs to recombine [24–27]. The X and Y PARs are therefore identical on average between males and females and are not thought to cause sex differences in phenotypes. The number and type of sex chromosomes present in females or males are referred to as the sex chromosome complement, and we use this term to encompass the parental imprinting and inactivation status of the X chromosomes (see below). For the purposes of this review, we break down the sex chromosome complement into a number of components that could contribute to sex differences in phenotypes. Aside from the PARs, which are present in two doses in female and male mice, the genomic dosage of these components is not the same in males and females (Table 1 and Fig. 1). Thus, females have two copies of the non-PAR region of the X chromosome (NPX), whereas males have one NPX and one non-PAR Y (NPY). Females also differ from males in that they receive an X chromosome from each parent, whereas males only receive an X from their mother. Because of parental imprinting, gene expression can differ between the maternal and paternal X chromosomes. Also, as is discussed more fully below, one of the X chromosomes in females is “inactivated.” Thus, about half of female cells inactivate the maternal X chromosome and experience the paternal X imprint (unlike any male cell), whereas the other half of the cells inactivate the paternal X chromosome and experience the maternal X imprint (similar to male cells). The inherent imbalance in the sex chromosome complement that results in a difference in gene expression between XX and XY cells can therefore be summarized as follows: (1) the absence vs. the presence of the NPY genes, (2) the difference in genomic dose of NPX genes (two vs. one copies), (3) any differences due to the parental imprint on the NPX genes, and (4) the presence vs. the absence of an inactive X chromosome (Table 1).

**Table 1:** XX vs. XY genomic dose for sex chromosome complement components

Genotypes	Gonads	NPX	NPY [−*Sry*]	*Sry*	PAR [−*Sts*]	*Sts*	X^m^	X^p^	X^i^
XX (40,XX)	F	2	0	0	2	2	1	1	1
XY (40,XY)	M	1	1	1	2	2	1	0	0

*Sry* and *Sts* are separated from the chromosomal regions that encompass them because mouse variants with sex chromosomes deleted for one or both of these genes are utilized in crosses

*NPX* non-PAR X genes, *NPY* non-PAR Y genes (excluding *Sry*), *PAR* pseudoautosomal region (excluding *Sts*), *X* ^*p*^ X chromosome of paternal origin, *X* ^*m*^ X chromosome of maternal origin, *X* ^*i*^ the inactive X which may be of maternal or paternal origin

Without any compensatory mechanism, the double genomic representation of X genes in XX cells, compared to the single representation of those genes in XY cells, would create an imbalance in X gene expression between the sexes (F>M); such a chromosome-wide sexual imbalance in expression is thought to be disadvantageous because X genes would not be in balance in both sexes with autosomal genes that they drive or that drive them [28]. X-inactivation, the process by which one X chromosome is largely transcriptionally silenced in non-germline XX cells, is an effective mechanism that reduces the imbalance in X gene expression. However, X-inactivation does not eliminate sex differences in the expression of X genes but reduces it to match approximately the level of sexual dimorphism in the expression of genes encoded on autosomes, which are present in two copies in each sex [29]. It also introduces the possibility of epigenetic effects of the inactive X, which are discussed more fully below.

X inactivation leads to mosaicism of X gene expression in XX mice. In eutherian mammals such as the mouse, the choice to inactivate the paternal or maternal X chromosome is random and patchy in tissues stemming from the inner cell mass of the blastocyst (i.e., the tissues giving rise to the body of the embryo), so that some somatic XX cells predominantly express X genes inherited from the mother, and others express X genes inherited from the father [30]. In outbred or natural populations that carry genetic polymorphisms, there are two types of X mosaicism—of the alleles and imprints. Because the maternally derived X chromosome will have many alleles that are different from those on the paternally derived X chromosome, roughly half of the cells in the XX mice will express the paternal X alleles and show the effects of any paternal imprint on X genes, and the other half will express the maternal X alleles and evince the effects of any maternal imprint on X genes. Thus, females are expected to have fewer extreme tissue phenotypes, because the extreme effects of specific X alleles (e.g., causing abnormal function) is often buffered by the expression of another allele in other cells of the same individual [31–33]. These effects are thought to lead to sex differences in the variability of tissue phenotypes (XX females less variable than XY males), and broader adaptiveness of female tissues to diverse environments because of the inherently greater diversity of X alleles within an individual female vs. an individual male. However, an overall reduced variability of X gene expression in human females, relative to males, has not been supported [34] but might be important under extreme environmental conditions or disease.

Thus, we can expand the list of possible sources of sex differences in phenotype arising from differences in sex chromosome complement, to include the following: (5) The inherent mosaicism of XX tissues arising from random X inactivation, which can lead to less phenotypic variability among females than males and to average differences in phenotype between the sexes; (6) sex differences in the prevalence of specific X alleles that may also lead to average phenotypic differences between the sexes. However, when studying inbred mice, all X alleles are the same in the two sexes, so these additional sources of sex differences are not operative.

Finally, it is possible that the difference in sex chromosome complement between normal males and females can lead to direct SCEs that are linked to the epigenetic status of the sex chromosomes rather than differences in X and Y gene expression [35–38]. One example of this arises from the fact that in somatic cells, the inactive X (X^i^) in females is rich in heterochromatin, reflecting the limited transcriptional activity. Heterochromatinization depends on enzymes that introduce repressive marks on histones, and it is hypothesized that heterochromatinization of the X competes with the introduction of repressive marks at some autosomal loci, thus affecting autosomal gene expression in females. This could be linked to a direct SCE even though it is not linked to an XX vs. XY difference in X gene expression. We do not consider this type of epigenetic contribution to direct SCEs further.

## Two mouse models that identify direct SCEs

An ideal mouse model for identifying direct SCEs is one that provides mice that differ in their sex chromosome complement (XX vs. XY) but not in the level of gonadal secretions. In practice, this is an elusive goal, but some experimental paradigms are informative even if strict control of gonadal hormone levels may not always be possible.

### Sf1 KO mice

One approach to eliminating the hormonal confound is to study mice that lack gonads. The most useful gonadless mice to date are those lacking steroidogenic factor 1 (*Sf1*, also known as *Nr5a1* and *Ad4bp*). *Sf1*-null mice of both sexes are born without gonads or adrenals and have a malformed hypothalamus [39, 40]. They die at birth because of the lack of glucocorticoids but are kept alive by injections of corticosterone and transplantation of adrenals [41, 42]. Such gonadless mice show XX vs. XY differences in some neural phenotypes, indicating that some sex differences, seen in gonadally intact mice in the same litters, are caused by direct non-gonadal effects arising from the difference in sex chromosome complement [43]. This model is valuable because it compares XX and XY mice or tissues that have never been exposed to gonadal secretions. However, the model has two main disadvantages: the homozygous *Sf1*-null mice are produced from heterozygous null parents, so that each litter produces a relatively small number of homozygous null pups, and each homozygous mouse must receive an adrenal transplant. We do not consider this model further.

### “Four core genotypes” (FCG) mice

The FCG model [44], in which the *Sry* gene causing testis development has been “moved” from the Y chromosome to an autosome, is so far the model most often used for separating the effects of sex chromosome complement from the effects of gonadal type. In general, the model can be used to identify sex differences caused by gonadal hormones (testicular vs. ovarian secretions) independently of sex chromosome complement (aside from *Sry*) and to identify sex differences caused by the direct effects of sex chromosome complement on non-gonadal tissues. The essence of this model is that the Y chromosome (here designated Yˉ) lacks the small region encoding *Sry,* and a functional *Sry* transgene has been introduced onto an autosome. XYˉ mice possessing the *Sry* transgene (XYˉ*Sry* mice) have testes and are fertile (XYM). Because *Sry* now segregates independently of the sex chromosomes, crossing XYM with normal XX female mice yields the four core genotypes: XX mice with ovaries (XXF) or testes (XXM), and XY mice with ovaries (XYF) or testes (XYM). Comparison of the FCG progeny is a 2 × 2 comparison of individuals that are either XX or XYˉ and have or lack the *Sry* transgene (Table 2).

**Table 2:** Progeny of FCG Cross XX x XYˉ*Sry* (XYM)

Genotypes	Gonads	NPX	NPY [−*Sry*]	*Sry*	PAR [−*Sts*]	*Sts*	X^m^	X^p^	X^i^
XXF	F	2	0	0	2	2	1	1	1
XXM	M	2	0	1	2	2	1	1	1
XYF	F	1	1	0	2	2	1	0	0
XYM	M	1	1	1	2	2	1	0	0

At the outset, it is important to establish that the XX vs. XY phenotypic sex difference of interest is seen in the FCG XXF vs. XYM comparison. Comparing the phenotype of mice with or without *Sry* is a measure of the effects of the *Sry* transgene: both the direct effects of *Sry* on tissues themselves and the indirect effects caused by testicular vs. ovarian secretions. Comparing XX and XYˉ mice allows assessment of the differential effects of sex chromosome complement (XX vs. XYˉ). Importantly, the comparison of XX and XYˉ in this model identifies differences that are common to the XXF vs. XYF and XXM vs. XYM comparisons and excludes differences found in normal XX females vs. XY males that are due to the *Sry* expression in males. The FCG does not separate the potential direct effects of *Sry* outside of the gonads from the indirect effects that are a consequence of *Sry* triggering testicular development, because the direct effects and hormonal effects are confounded (Table 2).

Although the FCG cross has been used successfully to identify a number of direct SCEs [12, 45, 46], investigators should be aware of the caveats considered in the next section.

## Important caveats relating to the FCG cross

### Genetic background

FCG mice have been generated on various genetic backgrounds—C57BL/6J inbred [47, 48], SJL inbred [49], C3H/He x C57BL/6 hybrid with a fixed B6 X [38], and “MF1” outbred with a fixed (“uniform”) MF1 X [50] (see “Genetic variability of NPX” section). However, with the C57BL/6J inbred FCG stock now available from the Jackson Laboratory (strain 010905) and difficulties with availability and maintenance of the F1 and outbred strains, the Jackson Laboratory strain is becoming the strain of choice.

### Potential differences in expression between endogenous and transgenic *Sry*

The key features of the FCG cross are (i) the deletion of *Sry* from the Y (this Yˉ, originally derived from the strain 129/SvEv-*Gpi1c*) and (ii) the *Sry* transgene [44]. The *Sry* transgene is located on chromosome 3 and is estimated to be present in 12–14 copies, although they may not all be functional [51]. Thus, the transgene may not have expression levels that are identical to those of the endogenous *Sry* encoded by the Y chromosome [52]; furthermore, there may be some ectopic expression relative to the endogenous *Sry*. If the differences between XX females and XY males depend on whether the *Sry* gene is wild-type (WT) or transgenic (as in FCG males)*,* then the FCG model could misrepresent the normal differences in WT XX vs. XY cells. However, the XX vs. XYˉ difference in the FCG model is assessed both in the presence and absence of the transgenic *Sry*, so that XX vs. XYˉ differences that occur under both conditions are likely to be robust and independent of *Sry* effects. To avoid the possibility of any abnormal effects of the transgene, some studies compare only XX and XYˉ females [53]. Furthermore, elements of the XX vs. XYˉ differences found in FCG mice (for example, the effects of one vs. two X chromosomes) can be potentially confirmed using the XY* model that has no transgene (see section on “Linking direct SCEs to a specific component(s) of the XX or XY complements” below) [47, 48].

### Genetic variability of NPX

When comparing the FCG genotypes, the NPX segments in XX and XY mice should be genetically identical, so that group differences attributed to sex chromosome complement (XX vs. XYˉ) are not confounded by NPX allelic differences. In inbred strains such as C57BL/6 (“B6”) or SJL, all the Xs are identical so the requirement for genetic uniformity of NPX is met.

### Differences in hormonal status in groups with the same type of gonad

Another issue for the FCG model is that the two groups of gonadal females and the two groups of gonadal males differ markedly in their fertility, which could be linked to hormonal differences. XYF on a B6 background are almost always sterile (PSB unpublished data), although on the MF1 outbred background, they do breed [54, 55]. Using the MF1 strain, it has been shown that it is a combination of a markedly reduced oocyte pool together with the expression of the Y-encoded transcription factor ZFY2 in the oocytes leading to impaired development of embryos prior to implantation, which severely limits the fertility [55]. Nevertheless, estrous cycle data collected in association with an FCG behavioral study [56] revealed that estrous cycles are still present in ~85–90% of MF1 XYF at 6–8 months of age (William Davies, personal communication), demonstrating that the majority of XYF ovaries are hormonally competent. FCG XYF on the B6 background have also recently been shown to cycle when assayed at 35–65 days of age and have similar levels of estradiol to XXF. However, ovaries are smaller in XYF than in XXF, and gonadotrophin levels are elevated in XYF relative to XXF especially after day 65, suggesting ovarian dysfunction or premature failure in XYF relative to XXF [9].

XXM are sterile with very small testes. This is because the presence of two X chromosomes in the germ cells leads to early spermatogenic failure [57, 58]. Nevertheless, XXM testes secrete androgens and the levels in XXM and XYM have been reported to be equivalent in adults [9, 52, 59, 60]; furthermore, numerous traits sensitive to testosterone levels during development are similar in XXM and XYM [44, 51, 61, 62]. For example, the anogenital distance is the same in XXM and XYM mice, and smaller in XXF and XYF relative to males, suggesting that the prenatal androgen levels that masculinize anogenital distance are similar in the XXM and XYM [51]. XXM are also reported to have elevated levels of follicle-stimulating hormone (FSH) relative to XYM from before puberty to age 5 months [9]. However, numerous studies have compared levels of testosterone in adult XXM and XYM, or of estradiol in adult XXF and XYF, and none has uncovered differences in plasma gonadal steroid levels to date [9, 52, 59, 60, 63–65].

Despite evidence against XX vs. XY differences in levels of circulating gonadal hormones, it is impossible to rule out differences that might occur before birth or in environmental conditions (stress, disease) when hormone levels are not measured. What steps can be taken to avoid the potential confound between these hormone differences and sex chromosome complement differences? One approach that has been used is to gonadectomize the mice (with or without equivalent hormone replacement) so that the phenotype of interest can be measured in mice that have the same levels of gonadal hormones at the time of measurement. However, although this approach controls for acute (“activational”) effects of hormones, it does not control for long term (“organizational”) effects of hormones prior to the gonadectomy [66]. Nevertheless, the FCG model allows an independent assessment of the importance of organizational effects of gonadal hormones, because the comparison of gonadectomized males and females tests for the importance of permanent effects of testicular vs. ovarian secretions. In some cases, robust differences between gonadectomized XX and XY mice have been found in the absence of evidence of organizational hormonal effects [47, 48, 67, 68], so that it is difficult to attribute those XX vs. XY differences to within-sex differences in the levels of gonadal hormones. Investigators may wish to measure phenotypes of gonadally intact FCG mice to test the effects of sex chromosome complement in the presence of ovaries or testes but are encouraged to test gonadectomized mice as well (with or without treatment with equal levels of gonadal hormone at the time of testing) to attempt to control hormone levels experimentally to better understand their role (see “FCG outcomes” section).

## FCG outcomes

**Fig. 2:**
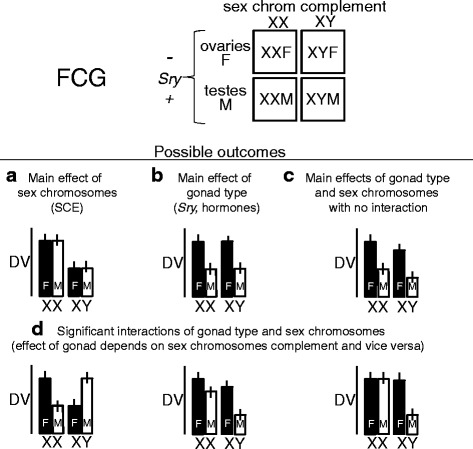
Possible outcomes of experiments using the four core genotypes to assess the effects of sex chromosome complement (XX vs. XYˉ) and gonadal sex (males *M* vs. females *F*) on a dependent variable (*DV*)

In what follows, we define *sex difference* to be a difference in phenotype between WT XX females and WT XY males that has been confirmed to be present in the FCG XXF vs. XYM comparison. The four outcomes correspond to outcomes of a 2 × 2 ANOVA with factors of *Sry* (absent or present, same as gonadal female vs. male) and sex chromosome complement (XX vs. XY) (Fig. 2).

1. An XX vs. XYˉ difference in phenotype occurs in both gonadal females and gonadal males (Fig. 2a), which is equivalent to the original sex difference found in WT mice. This outcome in the ANOVA is a main effect of sex chromosome complement in the absence of a main effect of *Sry* or an interaction. This result suggests that the sex difference is due to a direct SCE that is independent of *Sry*/gonadal secretions. Although this conclusion is satisfactory by itself, further tests are prudent, both to confirm the direct SCE in a different model (e.g., in XY* progeny, see next section on “Linking direct SCEs to a specific component(s) of the XX or XY complements”) or to test further for effects of gonadal hormones to rule them out. For example, it is possible to gonadectomize mice with or without hormone replacement, to rule out subtle hormonal confounds. Sex differences are rarely totally unaffected by gonadal hormones.
2. A phenotypic difference is found between mice differing in *Sry*/gonadal type, which is irrespective of sex chromosome complement and equivalent to the original sex difference found in WT mice. In the two-way ANOVA, this is a main effect of *Sry*/gonadal type in the absence of effects of sex chromosome complement (Fig. 2b). This result implies that the original sex difference is exclusively an effect of gonadal type (testicular vs. ovarian secretions) and/or a direct effect of *Sry* acting outside the gonads. Gonadectomy of FCG mice will confirm whether or not there is hormonal involvement and can help differentiate between activational and organizational hormone effects [67, 69].
3. The sex difference is independently influenced by *Sry*/gonadal type and by a direct SCE. This result leads to a significant main effect of both factors in the two-way ANOVA without a significant interaction (Fig. 2c). To investigate the hormones involved, and their times of action, one would perform further studies to manipulate the levels of gonadal hormones in both sexes either in adulthood or during early phases of development. A number of organizational effects are known to occur postnatally and thus can be revealed by gonadectomy, and prenatal organizational effects may be documented by manipulating hormone levels in utero.
4. The sex difference is caused by an interaction of sex chromosome complement and *Sry*/gonadal type, as is shown by a statistically significant interaction of the two factors in the two-way ANOVA (Fig. 2d) [47]. This result means that both factors contribute to the sex difference but that the effect of each factor depends on the level of the other factor (e.g., the effect of testicular secretions depends on whether the mouse is XX or XYˉ). Further experiments (gonadectomy, hormone treatments, etc.) would define the hormones involved and their times and sites of action in XX vs. XYˉ mice, and mechanistic experiments would define the molecular pathways influenced by both factors.

## Linking direct SCEs to a specific component(s) of the XX or XY complements

The strategies outlined below are based on the assumption that the direct SCEs are a consequence of differences in gene expression resulting from the difference in sex chromosome complement. If the tissue underlying the direct SCE can be identified with a high degree of confidence, then a case may be made for using RNA sequencing (RNAseq) on this tissue from the FCG in order to provide a list of candidate genes at this stage (see sections below beginning with “Candidate genes for direct SCEs). We will refer to this tissue as the “target tissue.” The candidate genes will be either on the NPX or NPY. The following crosses provide a logical approach to determining the extent to which NPX and/or NPY genes are responsible for direct SCEs.

Important: see Additional file 1, which provides the details of the three-generation breeding strategy needed to produce the fathers for Cross **B**. This involves progeny from Cross **C**.

### Identifying NPX and NPY effects using Cross **A**: XX♀ x XY*♂

**Fig. 3:**
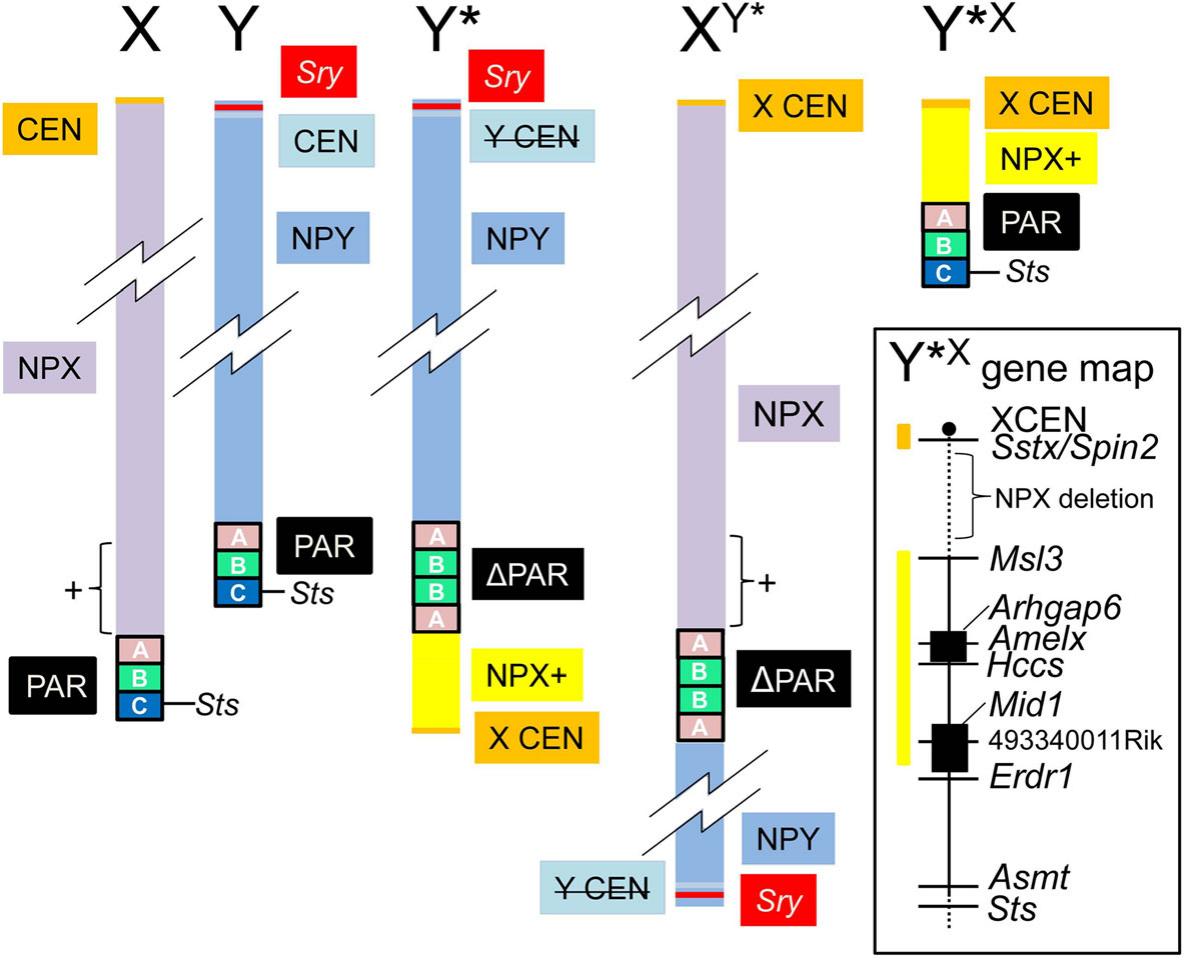
Sex chromosome structure diagrams related to Cross **A** (XY* model [70]). Sections of the sex chromosomes have been deleted to allow expansion of other regions for illustration. For the WT sex chromosomes, the PAR is illustrated in three sections^†^, one of which contains *Sts*. The minute + subregion of the NPX (<1% of the total NPX), adjacent to the PAR, is shown as a *bracketed region* within the entire NPX for the X and XX^Y*^ chromosomes, and in *yellow* for the Y* and Y*^X^ chromosomes. The Y* chromosome has an X centromere and the *plus* (+) region of NPX adjacent to a unique duplicated PAR that nevertheless lacks *Sts* (ΔPAR). The X centromere (X CEN) is the functional centromere, whereas the Y centromere is inactive (Y CEN)*.* The Y* chromosome undergoes abnormal recombination with the X chromosome to produce (1) the long X^Y*^ chromosome that is an end-to-end fusion of NPX and NPY with the ΔPAR lacking *Sts* and (2) the minute Y*^X^ chromosome with a normal PAR and the *plus* (+) region of NPX. The gene map for the Y*^X^ illustrates the approximate positions of genes transcribed from the forward strand (*black rectangles*) or reverse strand (*lines*), with the centromere (*orange*) and the *plus* (+) region of NPX (*yellow*) color coded. In effect, Y*^X^ is an X chromosome with a massive NPX deletion running from a region thought to be just proximal to *Msl3* and ending within the *Sstx*/*Spin2* multi-copy cluster adjacent to the telomeric X centromere [22, 72, 118–120]. The dividing line between NPX+ and the rest of NPX is not known precisely but is on the centromeric side of *Msl3* [72]. This figure updates and corrects earlier versions [50, 121]. ^†^ The PAR sections *A*–*C* were originally defined by mapping using the XY* model [71]: *A*, the terminal section of *Mid1*/*Fxy* (including the last three exons) that lies within the PAR [122]; *B*, the region that contains the multi-copy locus DXYHgu1 [123]; and *C*, the region containing *Sts* [124]. It is now known that *Erdr1* is located in the PAR just distal to *Mid1* and is retained in Y* and X^Y*^—thus, it maps to the PAR region B [125]. On the other hand, *Asmt* is absent in Y* and X^Y*^—thus, it maps to the PAR region C [126]. *Sts* is thought to be distal to *Asmt*, but this has not been confirmed

This cross is the best starting point for identifying potential NPX and NPY effects. The fathers have a variant Y chromosome (denoted Y*) that leads to the generation of a minute X chromosome derivative (termed Y*^X^ for historical reasons) composed of a complete PAR, no NPY, and a small subsegment of NPX (NPX+) currently estimated to be less than 1% of the total NPX (Table 3, Fig. 3) [70, 71]. As we shall see, the resulting X^m^Y*^X^ female progeny are used for Cross **C** that enables NPX dosage effects to be distinguished from NPX parental imprinting effects.

**Table 3:** Progeny of Cross **A**: XX x XY*

Genotypes^a^	Gonads	NPX^b^	NPY	*Sry*	PAR [−*Sts*]^b^	*Sts*	X^m^	X^p^	X^i^
X^m^O (rare)^c^	F	1	0	0	1	1	1	0	0
X^m^Y*^X^	F	1+	0	0	2	2	1	0	0
X^m^X^p^	F	2	0	0	2	2	1	1	1
X^m^Y*	M	1+	1	1	3	1	1	0	0
X^m^X^pY*^	M	2	1	1	3	1	1	1	1

^a^Parental sources of the X chromosomes (X^m^: maternal; X^p^: paternal) are included in Tables 4, 5, and 6, since these crosses are also used to identify effects of parental X imprinting

^b^Figure 3 illustrates the structures of the Y*, X^Y*^, and Y*^X^ chromosomes and the gene content of Y*^X^ that includes the minute + subsegment of NPX that is also present in Y*. Note that the Y* and X^Y*^ PARs have a duplication of PAR regions A and B but lack PAR region C that encompasses the *Sts* locus

^c^X^m^O are rare (~1% [75])

1. *Identifying an effect of NPX* (*two vs. one copies*).
  A comparison of X^m^X^p^ vs. X^m^Y*^X^ is a test of the effect of the number of NPX segments (2 vs. 1), keeping the number of PARs = 2.
  Cross **A** also tests for an NPX effect in the comparison of the two gonadal male groups, X^m^X^pY*^ with X^m^Y*, although in this case, the comparison deviates from the precise genetic conditions found in the XX-XY comparison in three ways: the presence of NPX+ in X^m^Y*; single copy of *Sts* in X^m^Y* instead of two copies found in XX or XY; and three copies of the region of the PAR that does not contain *Sts,* instead of two in XX or XY (Table 3). If the phenotypic difference between females containing one vs. two copies of NPX is similar to the difference between males with one vs. two copies of NPX, these deviations from normal XX and XY are generally considered minor [47].
2. *Identifying an NPY effect.*
  The effect of NPY can be detected in a comparison of X^m^Y*^X^ vs. X^m^Y* and X^m^X^p^ vs. X^m^X^pY*^. These comparisons are confounded with differences in the number of PAR segments as shown in Table 3. The Y* also has a different strain origin (LT/Sv) from that of the Yˉ from the FCG (strain 129/SvEv-*Gpi1c*). Thus, if the investigator finds no effect of the Y chromosome in this cross, it could be a Y chromosome strain difference. On the other hand, if a Y effect is found, then one can conclude that at least some Y chromosomes create a difference relative to XY*^X^ mice, which therefore may explain the original sex chromosome effect in FCG. Because the Y* chromosome encodes *Sry*, the NPY effect could be the result of testicular secretions; that possibility will already have been evaluated by a study of FCG mice, which will indicate whether the phenotype is influenced by gonadal hormones or not. If Cross **A** is used without knowledge of the outcomes from studies of FCG mice, it will not be possible to eliminate the hormonal confound of the Y* chromosome.
3. *Interpretation of outcomes*
  - NPX copy number effect only. The different effects of one vs. two copies of NPX can be caused by dosage differences (inherently higher expression of genes that escape X inactivation in XX than XY) or by X imprinting effects (different expression levels of paternally vs. maternally imprinted X chromosomes). Importantly, this cross tests for an effect of NPX copy number both in gonadal males (with *Sry*) and gonadal females (without *Sry*). If an NPX copy number effect is found in Cross **A** and in FCG mice, the results indicate that the effect does not depend on the *Sry* transgene in the FCG model.
  - NPY effect only. The SCE is attributed to NPY genes. Numerous NPY genes have an X “partner” gene that theoretically has a similar function (see section on “Candidate genes for direct SCEs” below)*.* If the NPY effect is caused by one of these genes, the implication is that the NPY gene has an effect distinct from its X partner.
  - NPX and NPY effects. In this case, it is possible that X and Y partner genes are involved, with the X gene escaping X inactivation [50]. Alternatively or additionally, the NPX effect could be due to imprinting.
4. *Advantages and drawbacks*
  - This a relatively simple cross that enables comparisons to be made quickly and economically.
  - Some of the X^m^Y*^X^ female offspring can be utilized as mothers for Cross **C** that enables parental X imprinting effects to be identified.
  - It has the minor disadvantage that the Y* chromosome has a different strain origin to that in the FCG cross.
  - It has the disadvantage that Y* encodes *Sry*, and thus all NPY-bearing mice are gonadal males, and differ hormonally from gonadal females that lack a Y chromosome. Thus, it does not test for an effect of NPY independent of its effect on gonadal secretions, unless prior studies of FCG mice indicate that the phenotype is not sensitive to gonadal hormones. This disadvantage is remedied in Cross **B**.
  - NPX is subject to X inactivation in X^m^X^p^. A minor concern is that in X^m^Y*^X^ the small NPX+ segment present on Y*^X^ is exempt from X inactivation because it does not include *Xist*. Genes in this segment are therefore expected to be expressed from both the X and Y*^X^ chromosomes [72]. In contrast, the same segment in X^m^X^p^ is expected to undergo random X inactivation and thus be expressed only from the active X chromosome. If these genes are expressed in the target tissue of the direct SCE, it is therefore likely that they will be expressed higher in XY*^X^ than XX. However, only a few known genes are encoded by NPX+ (Fig. 3) and they can be discounted if these genes show no difference in expression in the relevant target tissue.

### Identifying NPX and NPY effects using Cross **B**: XX♀ x XYˉY*^X^*Sry* ♂

As we have seen, there are a number of drawbacks to Cross **A** when it is used to attribute direct SCEs to NPX or NPY. This is particularly the case when the FCG outcomes indicate that gonadal hormones affect the direct SCE [50]. Cross **B** provides a more sophisticated assessment of potential NPX and NPY effects by bringing together the Yˉ chromosome and *Sry* transgene from the FCG cross, with the Y*^X^ chromosome generated by Cross **A**. Cross **B** is useful for balancing the number of PARs when assessing the effect of NPX dose, for example, in the comparison XX vs. XY*^X^. The cross produces the seven genotypes shown in Table 4, each with or without the *Sry* transgene, thus 14 genotypes in all. However, it is the three female/male pairs of genotypes with two sex chromosomes that are of primary interest. (The fathers for this cross are generated by mating X^m^X^p^Y*^X^ females from Cross **C** to XYˉ*Sry* males—see Additional file 1).

**Table 4:** Progeny of Cross **B**: XX x XYˉY*^X^ *Sry*

Genotypes^a^	Gonads	NPX^b^	NPY [−*Sry*]	*Sry*	PAR [−*Sts*]	*Sts*	X^m^	X^p^	X^i^
[X^m^O rare]	F or M	1	0	0F or 1M	1	1	1	0	0
X^m^Y*^x^	F or M	1+	0	0F or 1M	2	2	1	0	0
X^m^X^p^	F or M	2	0	0F or 1M	2	2	1	1	1
X^m^Yˉ	F or M	1	1	0F or 1M	2	2	1	0	0
[X^m^X^p^Y*^X^]	F or M	2+	0	0F or 1M	3	3	1	1	1
[X^m^YˉY*^X^]	F or M	1+	1	0F or 1M	3	3	1	0	0
[X^m^X^p^Yˉ]	F or M	2	1	0F or 1M	3	3	1	1	1

^a^Each genotype can be with or without *Sry*. Parental sources of the X chromosomes are indicated (X^m^: maternal; X^p^: paternal)

^b^Figure 3 includes the structure and gene content of the Y*^X^ chromosome

Cross **B** allows the following comparisons or interpretations:

1. *Confirming the sex chromosome complement effect identified in the FCG cross*
  The offspring of this cross include all the genotypes from the FCG cross, so the outcomes relating to X^m^X^p^ (F or M) vs. X^m^Yˉ (F or M) should be replicated here.
2. *Identifying an effect of NPX* (*two vs. one copies*)
  A comparison of X^m^X^p^ vs. X^m^Y*^X^ is a within-sex test (i.e., the comparison can be made either in gonadal females or in gonadal males) of the effect of the number of NPX segments (two vs. one), keeping the number of PARs = 2.
3. *Identifying an NPY* [*−Sry*] *effect.*
  A comparison of X^m^Y*^X^ vs. X^m^Yˉ provides a within-sex test of the effect of NPY [−*Sry*], keeping the number of PARs = 2.
4. *Interpretation of outcomes*
  - NPX copy number effect only. The different effects of one vs. two copies of NPX can be caused by dosage differences (inherently higher expression of genes that escape X inactivation in XX than XY), or by X imprinting effects, as for Cross **A**. Importantly, this cross tests for an effect of NPX copy number both in gonadal males (with *Sry*) and in gonadal females (without *Sry*).
  - NPY [−*Sry*] effect only. The SCE is attributed to NPY genes. Many NPY genes have an X partner gene that theoretically has a similar function (see section on “Candidate genes for direct SCEs” below). If the NPY effect is caused by one of these genes, the implication is that the NPY gene has an effect distinct from its X partner.
  - NPX and NPY effects. In this case, it is possible that X and Y partner genes are involved, with the X gene escaping X inactivation [50]. Alternatively or additionally, the NPX effect could be due to imprinting.
5. *Advantages and drawbacks*
  - The fathers for this cross can be bred by mating X^m^X^p^Y*^X^ females from Cross **C** to XYˉ*Sry* (FCG) males (Additional file 1).
  - This cross allows an elegant comparison of mice with two X chromosomes, one X chromosome, or one X and one Y chromosome (all with two PARs), each of which are produced as males or females. It is the only cross to achieve this degree of balancing of sex chromosome complement and gonadal sex. It also allows more comparisons than any other cross, to assess effects of NPX number, NPY, and gonadal sex.
  - Unlike Cross A, in this cross, NPY effects are not confounded by gonadal sex, and thus hormonal and NPY effects are more easily separated. NPY effects can be detected that occur in both gonadal males and gonadal females.
  - With 14 possible genotypes, the breeding is time-consuming and expensive.
  - Differentiating among the genotypes is difficult using chromosome spreads because the Y*^X^ is minute and easily missed; quantitative genomic PCR can confirm the presence or absence of Y*^X^ [73, 74] (Additional file 2).
  - As for Cross A, a minor concern when detecting an NPX effect is that the small NPX+ segment in X^m^Y*^X^ is present on both sex chromosomes but does not undergo X inactivation, in contrast to the same segment in X^m^X^p^ that is expected to undergo random X inactivation. If the NPX+ genes are expressed in the target tissue of the direct SCE, it is likely that they will be expressed higher in XY*^X^ than in XX. Similarly, when detecting an NPY effect, the absence vs. presence of NPY [−*Sry*] is confounded with the presence vs. absence of NPX+ (Fig. 3). However, NPX+ encodes only a small number of genes (Fig. 3) and they can be discounted if these genes show no difference in the expression in the relevant target tissue.

### Detecting effects of X chromosome imprinting

An NPX copy number effect identified by Cross **A** or **B** can be due to X genes that are exempt from the transcriptional silencing of one X due to random X inactivation [28]. In this case, transcript levels for these X genes are higher in X^m^X^p^ than in X^m^Y. An NPX copy number effect can also be due to parental X imprinting (see section on “Candidate genes for direct SCEs” below). For some X genes, this results in higher transcription from the X^p^ and thus elevated transcription in X^m^X^p^ relative to X^m^Y (an expression pattern similar to that for X genes that escape X inactivation); for other imprinted X genes, it results in higher transcription from the X^m^ and thus elevated transcription in X^m^Y relative to X^m^X^p^. However, in either case, X^m^/X^p^ transcript ratios in the X^m^X^p^ females can vary from one tissue sample to another if there is patchiness of random X inactivation in the target tissue sample (as exemplified in [30]). Here, we compare crosses that can identify effects arising from X imprinting and that compare female X^m^Y*^X^ (Cross **A**, Table 3) vs. X^p^Y*^X^ (Cross **C**, Table 5) in order to avoid the inherent variability of X^m^ vs. X^p^ expression associated with random X inactivation in X^m^X^p^.

**Table 5:** Progeny of Cross **C**: XY*^X^ x XY

Genotypes^a^	Gonads	NPX^b^	NPY [−*Sry*]	*Sry*	PAR [−*Sts*]	*Sts*	X^m^	X^p^	X^i^
[X^p^O]^c^	F	1	0	0	1	1	0	1	0
X^p^Y*^x^	F	1+	0	0	2	2	0	1	0
X^m^X^p^	F	2	0	0	2	2	1	1	1
X^m^Y	M	1	1	1	2	2	1	0	0
[X^m^X^p^Y*^X^]^d^	F	2+	0	0	3	3	1	1	1
[X^m^YY*^X^]	M	1+	1	1	3	3	1	0	0

^a^Parental sources of the X chromosomes are indicated (X^m^: maternal; X^p^: paternal)

^b^Figure 3 includes the structure and gene content of the Y*^X^ chromosome

^c^On a B6 background, these females die in utero [75]

^d^These X^m^X^p^Y*^X^ females are mated to XYˉ*Sry* males in order to produce the XYˉY*^X^ *Sry* males used for Cross **B** (see Additional file 1)

An effect of imprinting on X genes is detected by comparing female offspring of Cross A (XX x XY*) with those of Cross C (XY* x XY). As we have seen (Table 3), Cross **A** generates X^m^Y*^X^ females with a maternally imprinted X, together with X^m^X^p^ female controls. In Cross **C** (Table 5), the X^m^Y*^X^ females from Cross **A** are mated to XY males to produce X^p^Y*^X^ females with a paternally imprinted X, together with X^m^X^p^ female controls. For reasons that do not concern us here, Cross **C** also generates some X^m^X^p^Y*^X^ females and X^m^YY*^X^ males (Table 5). The important difference in Cross **C** relative to Cross **A** is that the XY*^X^ progeny carry a paternal X rather than a maternal X. This comparison is of mice that differ genetically only in the imprint on the single X chromosome.

1. *Detecting X* ^*p*^ *vs. X* ^*m*^ *imprinting effects*
  If an NPX effect detected in Cross **A** is at least in part caused by a parental imprint of the X chromosome, then a similar or enhanced difference should be detected when comparing X^m^Y*^X^ from Cross **A** with X^p^Y*^X^ from Cross **C**.
2. *Checking for maternal effects*
  Because these differences in imprint are potentially confounded with maternal effects (differences between crosses in maternal behavior, uterine effects, or other environmental differences), it is prudent to compare the X^m^X^p^ female progeny between the two crosses (**A** vs. **C**) as a check for confounding variables. If the XX females from the two crosses are similar, the confounding variables would seem not to be having a significant effect.
3. *Advantages and drawbacks*
  - These crosses have the advantage that they are both feasible on a B6 inbred background [75, 76]. As previously observed [75], from Cross **C**, there are no surviving X^p^Os, but ~11% of the offspring are X^p^Y*^X^ (Rhonda R. Voskuhl, personal communication).
  - Differentiating among the genotypes can be difficult using chromosome spreads because the Y*^X^ is minute and easily missed; quantitative genomic PCR can confirm the presence or absence of Y*^X^ [73, 74].
  - There is evidence from the study of embryos from XO females that maternal X monosomy has deleterious effects on development of preimplantation embryos [55, 77]; these effects could come into play in Cross **C**. See Additional file 3 for crosses **E** and **F** that avoid this potential confound.
  - X^p^O embryos are developmentally retarded in early pregnancy whereas X^m^O embryos are not [10, 75, 77–79]. This X^p^ effect would be expected to affect X^p^Y*^X^ embryos and thus is a potential confound when identifying imprinting effects. However, it appears to be ameliorated by the addition of Y*^X^.
  - The yield of X^p^Y*^x^ progeny in Cross **C** in B6 is low because of small litters. Probably because of the X^p^O death in utero, the X^m^Y*^X^ mothers often experience birthing problems leading to the death of viable X^p^Y*^X^ fetuses. A potential solution might be to set up timed B6 X^m^Y*^X^ x XY matings and timed F1 or outbred strain matings known to provide reliable foster mothers. Caesarian section and fostering can then be done when the B6 mothers are due to litter. There are various well-established humane husbandry protocols for this fostering procedure.

### Summary: a pragmatic approach

Taking into account the advantages and drawbacks discussed above, the most feasible strategy to investigate sex chromosome complement effects is as follows: (1) Establish a sex difference in gonadally intact XX females vs. XY males. The difference is most likely caused by the effects of gonadal hormones. (2) If experiments such as those recommended by Becker et al. [68] suggest that the sex difference is not caused entirely by differences in levels of gonadal hormones, one can use the FCG mice to detect sex chromosome complement effects that depend on, or are independent of, gonadal hormones. (3) If a sex chromosome complement effect is found in FCG mice, then Crosses **A** and/or **B** can be used to provide information as to whether the effect is caused by differences in NPX complement and/or the presence vs. absence of NPY. Cross **A** is easier than Cross **B**. Cross **B** is a more sophisticated cross for identifying NPX and NPY effects and is required to detect NPY effects if FCG outcomes make it difficult to separate hormonal and sex chromosome effects with Cross **A**. (4) Studies using Crosses **A** and **C** will assess the importance of X imprinting vs. NPX dosage. Together, these approaches will narrow down the X or Y candidate genes to be tested further as discussed in the next few sections.

## Candidate genes for direct SCEs

Here, we provide an overview of the sex chromosome genes whose differential expression between XX females and XY males may lead to direct SCEs in the target tissue in which the direct SCE originates. These comprise the following: (i) X genes that have parental imprints that lead to differential expression between males and females in the target tissue; (ii) X genes that escape X inactivation in the target tissue, an important group of which are X genes that have functionally similar partner genes on the Y chromosome (X-Y gene pairs that are termed “gametologs”); (iii) Y genes that are expressed in the target tissue of XY males.

### Parentally imprinted X genes

Classically, parentally imprinted genes were defined as those that were expressed only from the paternally derived chromosome or from the maternally derived chromosome. Recently, it has been established that parental imprinting can also lead to different levels of expression between the two parentally imprinted chromosomes. These two types of imprinting are now referred to as “canonical” and “noncanonical” imprinting, respectively [80]. There are two examples of canonical X gene imprinting that are relevant here. Firstly, there is the canonical imprinting of the *Xist* locus (reviewed by [32, 81]). This results in *Xist* only being expressed from the paternal X of XX embryos, beginning at the two-cell stage, which leads to the progressive inactivation of the paternal X during early cleavage stages. This paternal X inactivation is then removed from the blastocyst inner cell mass (the cells that form the body of the embryo) and is replaced with random *Xist*-mediated X inactivation, whereas the extra-embryonic tissues that contribute to the placenta retain paternal X inactivation [82, 83]. These *Xist* imprinting effects have to be borne in mind as a potential confounding factor. For example, paternal X imprinting leads to an early developmental retardation of X^p^O embryos, which might impact subsequent development [10, 78, 79, 84]. However, this retarding effect of Xp imprinting is much reduced in the context X^m^X^p^ female vs. X^m^Y male (or female) comparisons [8]. The only other canonically imprinted X genes that have been identified in mice are members of the *Xlr* gene family that are expressed from the maternal X; mice with only a paternal X exhibit impaired cognitive behavior [80, 85–87].

On the other hand, the study of Bonthuis et al. [80] identified 198 noncanonically imprinted X genes with 170 of such genes identified in a single tissue, the hypothalamic arcuate nucleus. In contrast to canonically imprinted genes, the imprinting was very tissue-specific. Clearly, this class of genes has the potential to make a significant contribution to X chromosome-associated direct SCEs. Intriguingly, there is a significant preponderance of X genes showing higher expression from the maternal X across all four tissues analyzed—see Figure S5A and D of Bonthuis et al. [80]. The impact of noncanonical imprinting on SCEs will increase as the expression from the two parental alleles becomes more disparate.

### X inactivation escapees

During the evolution of the X-Y chromosomes from their autosomal progenitors, there was a progressive loss of Y genes, while their X partners became subject to X inactivation to balance gene expression between males and females. However, a number of Y genes escaped attrition and the survivors are predominantly widely expressed regulatory genes. It is assumed that dosage sensitivity for these genes led to the retention of the Y copies because mutation of the Y gene was strongly deleterious so that that mutant Y chromosome was not passed on. Retention of the Y gene was associated with escape from inactivation of the X copies, thus retaining two expressing copies in males (X + Y) and females (X + X), preventing a deleterious level of expression relative to interacting autosomal genes [19, 20]. (See Table 6 for the X-Y gene pairs in mice and humans).

**Table 6:** Mouse and human Y genes and X gametologs

Class	Mouse					Human		
	Y gene	Copy no.	X gene	Xi?	Copy no.	Y gene	X gene	Xi?
Ancestral S1	*Sry*	1	*Sox3*	Yes	1	*SRY*	*SOX3*	Yes
	*Rbmy*	30	*Rbmx*	Yes	1	*RBMY*	*RBMX*	Yes
			[Aut.]			*HSFY*	*HSFX*	
			*Rps4*	Yes	1	*RPS4Y*	*RPS4X*	No
Ancestral S2	*Uba1y*	1	*Uba1*	Yes	1		*UBA1*	No
	*Kdm5d*	1	*Kdm5c*	No		*KDM5D*	*KDM5C*	No
	[*Tspy1ps*]		*Tspyl2*	Yes		*TSPY1*	*TSPX*	Yes
Ancestral S3	*Zfy1,2*	2	*Zfx*	Yes	1	*ZFY*	*ZFX*	No
	*Uty*	1	*Kdm6a*	No	1	*UTY*	*KDM6A*	No
	*Usp9y*	1	*Usp9x*	Yes	1	*USP9Y*	*USP9X*	No
	*Ddx3y*	1	*Ddx3x*	No	1	*DDX3Y*	*DDX3X*	No
	*Eif2s3y*	1	*Eif2s3x*	No	1	Note^a^	*EIF2S3X*	No
			*Amelx*	?	1	*AMELY* ^b^	*AMELX*	?
	Note^c^		*Eif1ax*	Yes		*EIF1AY*	*EIF1AX*	No
			*Tmsb4*	?		*TMSB4Y*	*TMSB4X*	No
			*Txlng*	?		*TXLNGY*	*CYorf15*	No
Ancestral S4/5			*Tbl1x*	Yes		*TBL1Y*	*TBL1X*	No
			[Aut.?]			*NLGN4Y*	*NLGN4X*	No
			[Aut.?]			*PRKY*	*PRKX*	No
Acquired^d^	*H2al2y*	2	*H2al2x*		14			
	*Prssly*	1						
	*Teyorf1*	1						
	*Rbm31y*	2	*Rbm31x*		1			
	*Sly*	126	*Slx,Slxl1*		39			
	*Ssty1,2*	306	*Sstx*		11			
	*Srsy*	197	*Srsx*		14			

Based on information from [19–22]

*Aut.* autosomal

^a^ *EIF2S3Y* function replaced by an autosomally located *EIF2S3X* retrogene [127]

^b^Ancestral S4 in [19]

^c^ *Eif1ay* function replaced by an autosomally located *Eif1ay* retrogene [127]

^d^Mouse data only

An important caveat is that the set of surviving Y genes varies among different groups of mammals. In mice, there seems to have been a tendency for the X copy of such gene pairs to become subject to X inactivation, and for the Y copy to diverge in sequence and acquire testis-specific functions (Tables 6 and 7). Also, the Y copies of the four X-Y gene pairs where the X is not subject to X inactivation are not essential in mice since X^m^O mice are viable; indeed, during fetal development, they are slightly ahead of XX fetuses and equivalent to XY fetuses [10]. This is in marked contrast to the lethality of non-mosaic human XOs [88]; this may reflect the larger number (14) of X inactivation escapees among X-Y gene pairs for which human XOs are dosage deficient [19]. Consequently, in mice, there are only four X partner genes that are clear X inactivation escapees as compared to 14 in humans.

**Table 7:** Mouse ancestral Y genes

Gene	Protein function	Expression^a^	Role [references]
*Sry*	HMG box transcription factor	Testis	Triggers the fetal genital ridge to form a testis [3, 98, 99]
*Rbmy,* ~30 copies	RNA binding motif protein	Testis; *brain*, *kidney*	Aids sperm morphogenesis [116, 128, 129]
*Uba1y*	Ubiquitin-activating enzyme	Testis; *brain*, *kidney*, *liver*, *skeletal muscle*	Not known [130, 131]
*Kdm5d* (*Smcy*)	Lysine specific demethylase	Ubiquitous	Has epigenetic effects by modifying histone H3. Interacts with MSH5 during spermatogenesis [132, 133]
*Zfy1,2*	Zinc finger transcription factors	Testis*, skeletal muscle*	Enable meiotic quality controls, the completion of the second meiotic division and sperm morphogenesis/function [73, 74, 104, 108, 109, 134]
*Uty*	Tetratricopeptide repeat protein without the demethylase activity of UTX/KDM6A (a demethylase)	Ubiquitous	Involved in protein-protein interactions? [98, 99, 105, 135]
*Usp9y*	Ubiquitin specific peptidase	*Testis*, *brain*, *kidney*, *skeletal muscle*	Loss of function in man leads to spermatogenic impairment [136, 137]
*Ddx3y*	Probable ATP-dependent RNA helicase	Ubiquitous	Not known [138]
*Eif2s3y*	Subunit of elongation and initiation factor	Ubiquitous	Involved in protein synthesis. Supports spermatogonial proliferation [100, 102, 139]

Data in italics indicate low transcript levels. For brain expression, also see [140]

^a^Based on RNAseq data [22]

Aside from these four X inactivation escapees that have Y partner genes, a number of other X genes show some degree of escape from X inactivation in vivo, and this escape can be tissue specific. The most recent study identified 34 of such genes, giving a total of 38 escapees—approximately 7% of mouse X genes [89].

### NPY genes

Because NPY genes are only expressed in males, any NPY gene could potentially contribute to a direct SCE that is identified using the FCG. A comprehensive compendium of the mouse Y gene content has recently been published [22]. Because FCG analyses often seek to shed light on human sex differences, in Table 7, we present the mouse Y gene complement alongside a subset of the human Y gene complement. The omitted human Y genes are those that have been “acquired” by the human Y but not by the mouse Y—for the most part, expression of these genes is restricted to spermatogenic cells [19, 20]. Similarly, the mouse has acquired genes that are not represented on the human Y, and the expression is thought to be restricted to spermatogenic cells; as such, they are unlikely to be involved in direct SCEs. Some of these genes are present in multiple copies and have multiple copy X gametologs. These genes are thought to have been amplified as a consequence of a post-meiotic X vs. Y genomic conflict [22, 23, 90]. The remainders of the mouse and human Y gene complements are termed “ancestral,” because they comprise a group of genes that were present on the Y of therian mammals at an early stage of Y chromosome evolution; all of these genes had gametologs on the X chromosome. Over time, some of the X and Y copies diverged, and in other cases, Y genes were lost differentially among mammalian groups. The mouse Y ancestral gene complement now comprises nine distinct genes, one of which (*Zfy*) has duplicated and one of which (*Rbmy*) is estimated to have 30 copies; the human Y has retained 17 ancestral genes, seven of which are also on the mouse Y. The nine ancestral mouse Y genes and the predicted characteristics of the proteins they encode are listed in Table 7.

### Identifying candidate genes that underlie direct SCEs

Key information for determining which genes might underlie a direct SCE is their expression profile. RNA sequencing (RNAseq) is the current method of choice for documenting the transcriptome in specific tissues or cell types. It has the potential to document the relative abundance of all the transcripts present in a tissue sample—the detection of rare transcripts can be improved by increasing the sequencing coverage depth. While RNAseq data for the relevant target tissue may be available for XY female vs. XY male comparisons, hormone effects may modulate or nullify expression differences linked to the direct SCE. RNAseq data from FCG mice would help to uncover such effects. In the following sections, we outline how such transcriptome information can be utilized to home in on the X and/or Y genes that generate the direct SCE.

## Identifying NPX genes that cause direct SCEs

Here, we consider strategies for identifying the genes underlying a direct SCE when NPX (but not NPY) has been implicated in Cross **A** or **B**. The first step is to use the FCG RNAseq data to generate a list of X genes that are differentially expressed in the target tissues of XXF vs. XYF and/or XXM vs*.* XYM FCG mice, under conditions in which the differences are unlikely to be caused by gonadal hormones.

As we have seen, the most abundant may well be X genes subject to non-canonical parental imprinting effects. If such genes are involved in generating the direct SCE, this should have been detected in the X^m^Y*^X^ vs. X^p^Y^X^ (Cross **A** vs. **C**) comparison in the section above entitled “Linking direct SCEs to a specific component(s) of the XX or XY complements”*.*

- *X gene transcript levels where X* ^*m*^ *Y > X* ^*m*^ *X* ^*p*^ (Table 8)*.* If the canonically imprinted *Xlr* genes are involved, they will fall into this category. Other X genes falling in this category are prime candidates for being X genes with noncanonical imprinting that leads to reduced expression of X^p^ relative to X^m^. This is the predominant form of noncanonical X gene imprinting (see Figure S5A and D in [80]).

**Table 8:** Predicted expression of imprinted X genes

	If X^p^ = Xi	If X^m^ = Xi	Predict X gene expression in crosses
Maternal expression (paternal imprint)	Expression high	Expression low	FCG: X^m^X^p^ < X^m^Y Cross A: X^m^Y*^X^ = X^m^Y* > X^m^X^p^ = X^m^X^pY*^Cross B: X^m^Y > X^m^X^p^ > X^p^Y*^X^Cross C: X^m^Y* = X^m^Y*^X^ > X^m^X^p^
Paternal expression (maternal imprint)	Expression low	Expression high	FCG: X^m^X^p^ > X^m^Y Cross A: X^m^Y*^X^ = X^m^Y* < X^m^X^p^ = X^m^X^pY*^ Cross B: X^m^Y < X^m^X^p^ < X^p^Y*^X^ Cross C: X^m^Y* = X^m^Y*^X^ < X^m^X^p^
- *X gene transcript levels where X* ^*m*^ *X* ^*p*^ *> X* ^*m*^ *Y* (Table 8). This could be due to (i) the noncanonical imprinting where there is greater expression of X^p^ relative to X^m^ or (ii) to genes that escape X inactivation, in particular, the four widely expressed mouse X inactivation escapees *Kdm5c/Smcx*, *Kdm6a/Utx*, *Ddx3x*, and *Eif2s3x* (Table 6) or the additional escapees identified by in vivo studies [89, 91]. The X escapees are among the genes most often found to differ in XX vs. XY mice in microarray-based transcriptome profiling on various adult tissues of FCG mice (Arnold, unpublished), and these have been repeatedly reported to be expressed higher in XX than in XY mice [47, 48, 72, 92–97].

The X genes that exhibit expression differences compatible with the direct SCE identified in the FCG mice would be prioritized for further study. The X genes might first be ranked with respect to the fold difference in the level of expression between the relevant FCG genotypes (e.g., XXF vs. XYF), and/or by the *p* value of a statistical test that estimates the reliability of the difference in the expression between groups. These candidate genes could be further prioritized based on their known characteristics and potential relevance to the phenotypes under investigation. If necessary, candidate genes would be checked using quantitative RT-PCR to confirm the expected group differences. If the candidate NPX gene is a reported X escapee, and is expressed higher in XX than in XY, as expected from its status as an escapee, its importance as a gene causal to the SCE would be established by comparing mice with one vs. two copies of the gene. For example, if *Ddx3x* (Table 6) is the candidate, one might compare WT XX mice (with two copies of *Ddx3x*) with XX mice with a heterozygous knockout of *Ddx3x* (one copy). If these groups of mice differ in the phenotype showing the SCE, such that one vs. two copies of *Ddx3x* mimics the phenotypic effects of one vs. two copies of NPX, then *Ddx3x* is a likely contributor to the SCE. This experiment determines if dosage differences in *Ddx3x* alone are *necessary* to show the SCE. An additional strategy is to add a transgene encoding *Ddx3x* to an XO or XY*^X^ mouse, to determine if the phenotype is affected appropriately in mice with one copy vs. more than one copy of the candidate X escapee. The transgenic add-in experiment aims to test if the dosage differences in the X escapee are *sufficient* by themselves to mimic the SCE. The main caveat is that expression levels of the transgene may be difficult to control depending on its promoter, position effects, etc.

The experiments comparing Cross **A** vs. **C** may suggest that the NPX effect is the result of imprinting. In this case, transcriptome profiling may reveal either XX > XY or XY > XX expression patterns (Table 8) and differences in expression of transcripts in X^m^Y*^X^ vs. X^p^Y*^X^. To provide further strong support for an NPX imprinting explanation of an SCE, one might measure the effects of parent of origin on the epigenetic status of the candidate gene and manipulate its expression to mimic the differences in expression found in X^m^Y*^X^ vs. X^p^Y*^X^, to demonstrate that these differences in expression alone are necessary and sufficient to mimic the SCE, at least in part.

The manipulation of NPX expression is best performed in a tissue-specific manner, assuming that the tissue of interest is known. For example, a floxed allele of the candidate gene can be knocked down with a tissue-specific Cre. Indeed, the use of tissue-specific knockdown or overexpression may help determine the tissue in which the candidate gene causes the SCE.

## Identifying NPY genes that cause direct SCEs

Here, we consider strategies for identifying the genes underlying a direct SCE when NPY (but not NPX) has been implicated in Cross **A** or **C**. The first step is to check the RNAseq data to provide a list of the Y genes expressed in the SCE target tissue. Prioritizing the candidate genes may be possible based on the current information about the function of the Y genes or their similar X gametologs.

Given that only seven Y genes (*Uba1y*, *Kdm5d*, *Uty*, *Usp9y*, *Ddx3y*, *Eif2s3y*, and *Ssty2*) have been shown to be widely expressed in non-gonadal tissues as assayed by RNAseq [22], the “prioritized list” of genes expressed in a target tissue with a putative NPY-linked SCE will likely be limited to one or a very few genes. Thus, it makes sense to move on to the stage of manipulating candidate Y gene expression in order to demonstrate that the SCE of interest is a consequence of the presence vs. the absence of a particular NPY gene or genes. The basic approach is the same as for candidate NPX genes discussed in the last section “Identifying NPX genes that cause direct SCEs”. Keeping other factors constant (e.g., sex chromosomes and hormones), the investigator varies the presence/absence of one candidate Y gene to determine if that manipulation causes a phenotypic difference similar to the SCE caused by the comparison of XX vs. XY. Two basic approaches are available, either knocking out the NPY gene in XY mice [98–101] and comparing the knockout (KO) to WT XY or adding a transgenic copy of the candidate NPY gene in mice with one X chromosome (e.g., XO or XY*^X^) and comparing to controls without the transgene [73, 102]. It would be best to start with global KO or transgenic, where the genetic manipulation affects all tissues, but tissue-specific gene targeting or transgenesis would ultimately be a powerful demonstration of the effect of the NPY in the target tissue. Transgenic lines encoding autosomal copes of NPY genes (*Uty, Kdm5d, Ddx3y, Usp9y, Ubely*, and *Eif2s3y*) were developed in the Burgoyne Lab and are being backcrossed to B6 in the Arnold Lab.

It is possible but less likely that multi-copy NPY genes account for the SCE. Manipulating these genes is more problematic. One option is to knock down the expression using a transgene that delivers small interfering RNAs such as has been successful for the multi-copy NPY gene *Sly* [103].

Investigators who have obtained evidence for NPY involvement might consider using crosses in Additional file 4 to map the NPY genes that underlie the direct SCE to specific regions of the Y. The first cross involves a commercially available *Sxr* ^*a*^ mutation that involves translocation of Y short arm (Yp) genes to the tip of the X or Y PAR and will establish if the SCE involves Yp genes that map to *Sxr* ^*a*^ [104]. If this is not the case, the second cross utilizing a Y deletion (Y^d1^) should provide confirmation that multi-copy NPY genes are involved. A contact e-mail is provided in Additional file 4 for those wishing to use this Y^d1^ deletion.

## Interpreting cases of dual NPX and NPY effects obtained with Cross **A** or **B**

Here, we consider the strategies for identifying the genes underlying a direct SCE when NPX and NPY have similar effects in Cross **A** and/or **B**. The prime candidate genes are the ancestral X-Y gene pairs *Uba1y/Uba1*, *Kdm5d/c*, *Zfy/x*, and *Uty/x* (*Utx* A.K.A. *Kdm6a*), *Usp9y/x*, *Ddx3y/x*, and *Eif2s3y/x;* Table 6). As discussed in the section above, “Candidate genes for direct SCEs”, the X partners of these gene pairs are widely expressed dosage-sensitive regulatory genes [19, 20]. The conservation on the Y chromosome of the Y partner gene during evolution is thought to have been driven by dosage balance of expression and function of the X and Y gametologs, which implies equivalent function of the two genes. However, over time, there were varying degrees of sequence divergence between the X and Y copies, with the Y copies often acquiring some specific functions. Thus, a central unresolved question is whether the X and Y partner genes have similar or divergent function in different tissues and life stages. Limited evidence to date indicates that the function of the X and Y genes outside of the testes is different in some cases such as *Uty/Utx* [105].

The RNAseq data provides a list of the X and Y genes that are expressed in the target tissue, so it is reasonable to prioritize any X-Y gene pairs. If any X genes are known to show linkage to the trait, they would have a high priority whether or not they are a member of an X-Y gene pair. For X-Y gene pairs, the relative abundance of X + X transcripts in XX females compared to X + Y transcript abundance in males are not very informative when the X and Y transcripts have diverged such that the encoded proteins are not equivalent in activity and/or function. Once again, the most direct approaches to identifying the X and Y genes involved are gene targeting and transgenesis.

Nevertheless, the possible balance of effects of X-Y pairs could lead to an unusual scenario, in which Crosses A and B show effects of NPX dose, or NPY dose, when in fact, the study of FCG mice shows no direct SCE. In this case, a likely scenario is that both members of an X-Y gene pair have effects on the phenotype but that the effects are balanced, and therefore produce no phenotypic difference in XX vs. XY. Similarly, when gene dose is manipulated through knockout of single copies of X-Y gene pairs, reducing the dose of both X and Y partners could show a phenotypic effect that is balanced and does not contribute to sex differences in phenotype. The balance of X and Y partner genes could be dynamic, however, changing under different life conditions (stress, disease, developmental stage, tissue type, and hormone levels) [50].

## Looking for direct SCEs when there is no sex difference

Most investigators will be drawn to the study of FCG mice if they hypothesize a direct SCE because classical experiments of hormone removal and replacement fail to explain a known sex difference in phenotype [69]. However, FCG mice can be informative even if a sex difference in the phenotype is not established. In some cases, two sex-biasing factors typical of one sex (e.g., female levels of estradiol and a second X chromosome [47]) can reduce or eliminate the effects of the other [46, 106]. Reducing the effects of one sex-biasing factor can therefore “unmask” the effects of another. The “compensatory” actions of hormones and direct SCEs provides a rationale for comparing groups of FCG mice even if there is no overt sex difference in a phenotype [106]. In published studies, FCG mice have often been gonadectomized to allow comparison of groups under conditions in which the levels of gonadal hormones are equal, to reduce the possibility of gonadal hormonal confounds for direct SCEs. Direct SCEs discovered in gonadectomized (GDX) mice are sometimes, however, reduced if the mice have gonads or are treated with hormones after GDX [107]. Direct SCEs that are smaller in the presence of gonads or gonadal hormones are potentially important because various life events (aging, stress, disease) can result in reduced levels of gonadal hormones, which can result in the emergence of direct SCEs that contribute to sex differences in physiology. The interaction of direct SCEs and hormones, when the effect of one sex-biased variable is conditioned by the level of the other factor, indicates that the two types of variables (sex chromosome genes, gonadal hormones) have convergent actions on gene pathways influencing emergent phenotypes including the disease [45]. The molecular nature of this interaction is unstudied to date.

## Conclusions

**Fig. 4:**
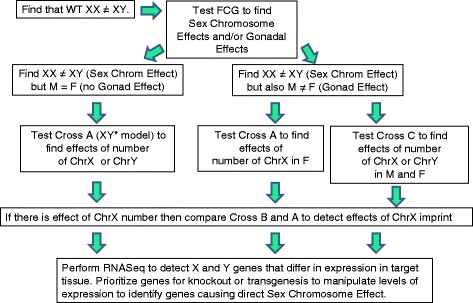
Logic tree for genetic dissection of direct sex chromosome effects

Our attempt here has been to provide the basis for a logical dissection of sex chromosome effects on any phenotype in mouse. The decision tree that summarizes this approach is shown in Fig. 4. Because the difference in the XX and XY genomes is fundamentally a difference in copy number of large segments of X and Y DNA, it makes sense to start by varying the number of these segments in combination or in isolation, to narrow down the segments that make XX and XY mice different. A progressive narrowing of choices can ultimately lead to the discovery of new genetic factors that make males and females different. The approaches suggested here have the advantage that they mimic the natural differences between XX and XY tissues. Other methods to search for X chromosome effects, for example, linkage mapping for specific traits, are not equivalent and may not detect differences caused by one vs. two X chromosomes. For example, study populations may not have any variation in the X genes that contribute to direct SCEs or variation in gene sequence may not mimic effects of X chromosome number. Similarly, studying the effects of different strain origin of the Y chromosome (e.g., by comparing consomic Y strains) may uncover effects of Y chromosome variation but may not mimic the effects of the presence vs. absence of the Y chromosome as it relates to sex differences in phenotype.

The approaches suggested here have already demonstrated that both X and Y segments cause differences in phenotype [11, 50]. Specific Y genes have been identified that control specific aspects of spermatogenesis [73, 102, 104, 108, 109], but to date, no specific X gene has been identified that causes a sex chromosome effect. Because of the advent of more efficient gene targeting methods, we expect this situation to change soon. It will be interesting to discover which X imprinted or escapee genes cause differences in physiology and disease, and how these and Y genes interact with gonadal hormones to cause emergent sex differences in phenotype.

## Additional files

Additional file 1: Obtaining parents for B6 crosses used in the “Linking direct SCEs to a specific component(s) of the XX or XY complements” section. (See Additional file 2 for genotyping protocols). (DOCX 15 kb)
Additional file 2: Progeny genotyping protocols relating to Additional file 1 [51, 72–74, 110, 111]. (DOCX 25 kb)
Additional file 3: Previously utilized MF1 crosses relevant to this review [10, 38, 67, 70, 71, 75, 77–79, 85, 112–115]. (DOCX 31 kb)
Additional file 4: Crosses with Y chromosome translocations or deletions. (See chromosome diagrams in [55, 104, 116, 117]). (DOCX 20 kb)

## Abbreviations

ANOVA: Analysis of variance
CEN: Centromere
F: Female
FCG: Four core genotypes mouse model
KO: Knockout
M: Male
NPX: Non-PAR region of the X chromosome
NPX+: Minute region of the NPX near the PAR
NPY: Non-PAR region of the Y chromosome
*Paf*: Patchy fur mutation
PAR: Pseudoautosomal region
RNAseq: RNA sequencing
SCE: Sex chromosome effect
WT: Wild-type
X^i^: Inactive X chromosome
X^m^: X chromosome inherited from the mother
X^p^: X chromosome inherited from the father
XXF: XX mice with female gonadal sex (developed ovaries)
XXM: XX mice with male gonadal sex (developed testes)
XYF: XY mice with female gonadal sex (developed ovaries)
XYM: XY mice with male gonadal sex (developed testes)
Y*: Specific variant of the Y chromosome with a normal NPY but abnormal PAR
Yˉ: Y chromosome deleted for *Sry*
