# Supplementary material for: A primer on the use of mouse models for identifying direct sex chromosome effects that cause sex differences in non-gonadal tissues
Source: Biol Sex Differ. 2016 Dec 13;7:68. doi: 10.1186/s13293-016-0115-5 (PMC5154145; doi:10.1186/s13293-016-0115-5)
Supplement: Additional file 1: — Obtaining parents for B6 crosses used in the “Linking direct SCEs to a specific component(s) of the XX or XY complements” section. (See Additional file 2 for genotyping protocols). (DOCX 15 kb) [file 13293_2016_115_MOESM1_ESM.docx]

**Additional file 1. Obtaining parents for B6 crosses used in section 5.** (See Additional file **2** for genotyping protocols)

We suggest the following mouse breeding strategy in order to generate the fathers for Cross **B**: (a) Set up matings for Cross **A**. (b) When mature, use XY*^X^ female progeny from Cross **A** to start some Cross **C** matings in order to generate some XXY*^X^ female progeny. (c) When mature, mate the XXY*^X^ females from Cross **C** to XYˉ*Sry* (FCG) males, to produce the XYˉY*^X^*Sry* fathers for Cross **B**.

Cross **A**: XX x XY*.

XY* mice are B6Ei.LT-Y(IsXPAR;Y)Ei/EiJ mouse stock 002021 from Jackson Laboratory, which has a strain background slightly different from C57BL/6J. In the Arnold lab this strain has been backcrossed for more than 10 generations to C57BL/6J.

| Progeny^1^ | Gonads | Comments |
| --- | --- | --- |
| [X^m^O] | F | X^m^O are rare particularly with B6 crosses. The remaining genotypes occur with approximately equal frequencies. |
| X^m^Y*^X^ | F |  |
| X^m^X^p^ | F |  |
| X^m^Y* | M |  |
| X^m^X^pY*^ | M |  |

^1^ X^m^ maternal; X^p^ paternal

Cross **C**: XY*^X^ x XY.

X^m^Y*^X^ females from cross **A** are mated to C57BL/6 XY males (Jackson Laboratory stock 000664).

| Progeny^1^ | Gonads | Comments |
| --- | --- | --- |
| [X^p^O] | F | The XY*^X^ mothers have small litters.  X^p^O are conceived but do not survive in B6 crosses. 11% of surviving offspring are X^p^Y*^X^.  The X^m^X^p^Y*^X^ females used for cross D comprise 34% of offspring (R.R. Voskuhl, pers. comm.). |
| X^p^Y*^X^ | F |  |
| X^m^X^p^ | F |  |
| X^m^X^p^Y*^X^ | F |  |
|  |  |  |

^1^ X^m^ maternal; X^p^ paternal. Only female progeny are listed.

Cross **D**: XXY*^X^ x XYˉ*Sry* (to produce fathers for Cross **B**).

XXY*^X^ females from Cross **C** are mated to FCG XYˉ*Sry* males (see sections 2-4). A C57BL/6J inbred FCG stock is available from the Jackson Laboratory (stock 010905).

| Progeny^1^ | Gonads | Comments |
| --- | --- | --- |
| X^m^X^p^*Sry* | M | The four male genotypes from this cross are expected to be produced in equal frequencies.  The X^m^YˉY*^X^*Sry* male progeny used as fathers for cross **B** (see section 5) are mated to C57BL/6J females (Jackson Laboratory stock 000664). |
| X^m^Yˉ*Sry* | M |  |
| X^m^X^p^Y*^X^*Sry* | M |  |
| X^m^YˉY*^X^*Sry* | M |  |
|  |  |  |

^1^ X^m^ maternal; X^p^ paternal. Only male progeny are listed.

Cross **B**: XX x XYˉY*^X^*Sry*

| Progeny^1^ | Gonads | Comments |
| --- | --- | --- |
| [X^m^O rare] | F or M | This Table lists the progeny generated by cross **B** (see Table 3). This is needed in the context of the genotyping protocols in **Additional file 2**. Importantly, in this cross and cross **D** above, male gonadal sex (M) is dependent on the presence of the *Sry* transgene. |
| X^m^Y*^x^ | F or M |  |
| X^m^X^p^ | F or M |  |
| X^m^Yˉ | F or M |  |
| [X^m^X^p^Y*^X^] | F or M |  |
| [X^m^YˉY*^X^] | F or M |  |
| [X^m^X^p^Yˉ] | F or M |  |

^1^X^m^: maternal; X^p^: paternal. Each genotype can be + or - *Sry*.
