# Supplementary material for: A primer on the use of mouse models for identifying direct sex chromosome effects that cause sex differences in non-gonadal tissues
Source: Biol Sex Differ. 2016 Dec 13;7:68. doi: 10.1186/s13293-016-0115-5 (PMC5154145; doi:10.1186/s13293-016-0115-5)
Supplement: Additional file 2: — Progeny genotyping protocols relating to Additional file 1 [51, 72–74, 110, 111]. (DOCX 25 kb) [file 13293_2016_115_MOESM2_ESM.docx]

**Additional file 2.** Progeny genotyping protocols relating to Additional file **1.**

1. **Gonadal sex and FCG PCR**

The first step for each cross is to assess the gonadal sex. Trained staff can determine this by examination of the anogenital distance soon after birth. For males, important further information can be gained from assessing testis size and fertility status (see the extensive literature relating to FCG mice). However, there is a reliable gonadal sex PCR that is applicable to all the crosses in Additional file 1. This utilizes PCRs for multiple *Ssty* copies on the Y chromosome long arm, the testis-determining gene *Sry,* and *Myog* (on chromosome 1) as a control (Additional Figure 1). In, for example, for the cross XX x XY*, the PCR for the Y chromosome marker *Ssty* is sufficient to identify gonadal males. However, in crosses involving the FCG Y^-^ that lacks *Sry*, it is necessary to also include *Sry* primers to detect the autosomally located *Sry* transgene. In practice it will be easiest to always run the triplex PCR, but bear in mind that the multiple copy *Sry* transgene in FCG males gives a much stronger band than endogenous *Sry* (see Figure 1 in [51]). This PCR utilizes the following conditions: 95^o^C for 4 min, followed by 35 cycles of 94^o^C for 45 sec, 58^o^C for 30 sec and 72^o^C for 1 min, with a final extension at 72^o^C for 5 min.
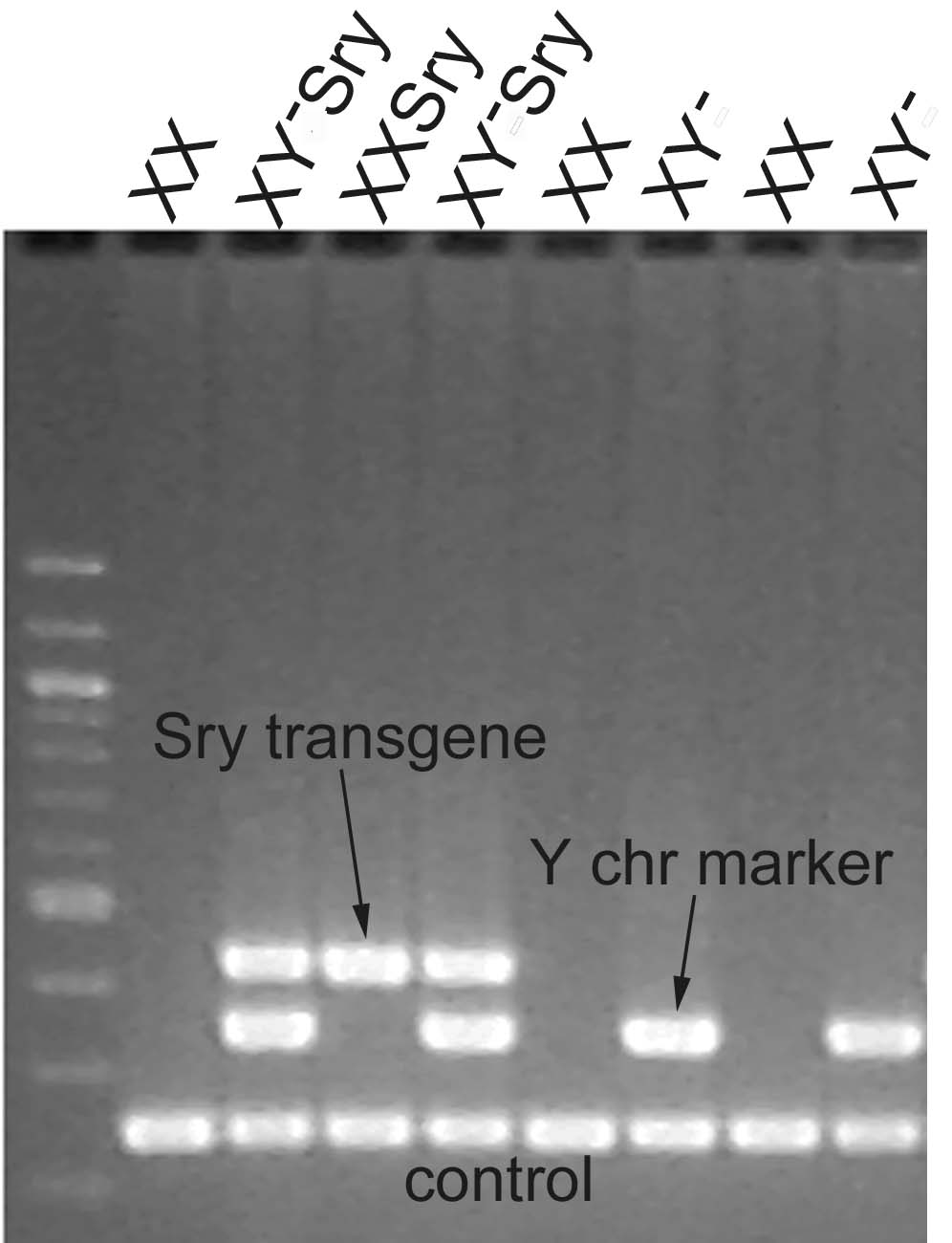


Additional Figure 1. Gel image of products from triplex genomic PCR to distringuish genotypes of the Four Core Genotypes. The three bands correspond to the *Sry* transgene (which determines male gonadal sex), the Y chromosome marker to detect presence of the Y chromosome, and myogenin autosomal gene which check that the PCR reaction worked. Size ladder is 100bp.

**List of primers**

| Gene | Primer ID | Primer sequence | Amplicon  size |  |
| --- | --- | --- | --- | --- |
|  | | | | |
| *Ssty* | *Ssty*‑F | CTGGAGCTCTACAGTGATGA | 342bp |  |
|  | *Ssty*‑R | CAGTTACCAATCAACACATCAC |  |  |
| *Sry* | *Sry*‑F | AGCCCTACAGCCACATGATA | 420bp |  |
|  | *Sry*‑R | GTCTTGCCTGTATGTGATGG |  |  |
| *Myog* | *Om1a*^1^ | TTACGTCCATCGTGGACAGCAT | 246bp |  |
|  | *Om1b*^1^ | TGGGCTGGGTGTTAGTCTTAT |  |  |
|  |  |  |  |  |

^1^The *Myogenin* PCR using *Om1* primers (see [110]) is a positive control that the PCR worked.

1. **NPX qPCR**

The second step is to identify the NPX dosage. The quantitative real time genomic PCR (qPCR) presented here is designed to distinguish between NPX dosage and the dosage of the minute NPX+ segment present in the Y* and Y*^X^ chromosomes (Additional Figure 2).

In order to discriminate between the different genotypes, a qPCR test has been developed using two X-linked genes: *Prdx4*, present only on the X chromosome, and *Amelx* located very close to the pseudo-autosomal region and thus present on the X, Y* and Y*^X^ chromosomes. An autosomal gene *Myogenin* (*Myog*, chromosome 1) is used for normalization of each *Prdxa* and *Amelx* PCR. The primers used are listed below.

**
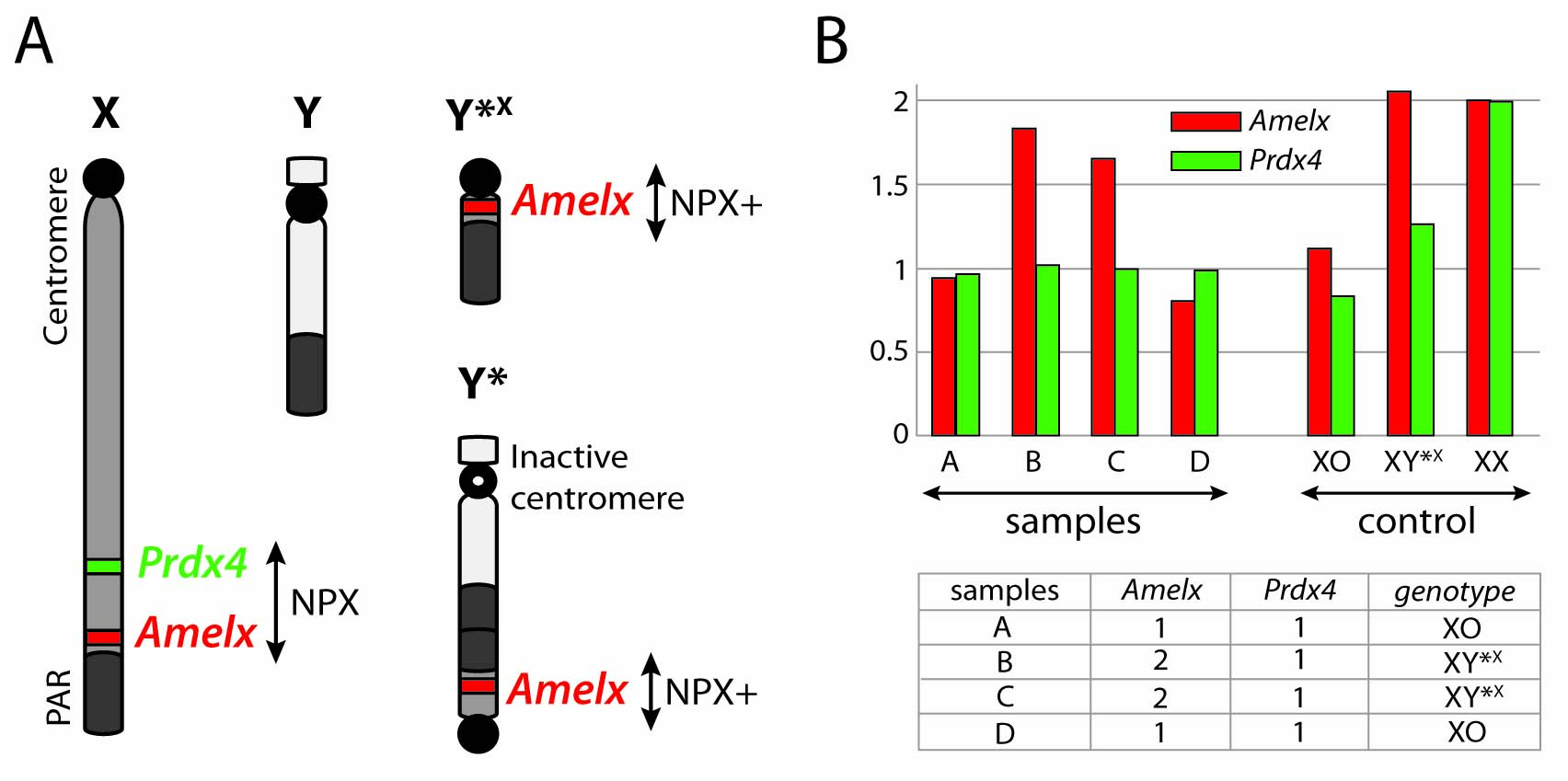
**

Additional Figure 2. Illustration of quantitative genomic PCR to distinguish between NPX dosage and dosage of the minute + segment of NPX (see Figure 3 and Table 3). A. The chromosomal diagrams show the location of the NPX gene *Pdrx4* and NPX+ segment gene *Amelx*. B. An example of an experimental outcome showing expression levels of the two genes in progeny of XY* fathers. An autosomal gene *Myogenin* (*Myog*, chromosome 1) is used for normalization of each *Prdxa* and *Amelx* PCR (not shown).

**List of primers**

| Gene | Primer ID | Primer sequence | Amplicon  size |  |
| --- | --- | --- | --- | --- |
|  | | | | |
| *Prdx4* | *Prdx4*‑F | CATGATATCCACTGAAAGCTAC | 82bp |  |
|  | *Prdx4*‑R | GAGACAGTGTATCTATCCCTG |  |  |
| *Amelx* | *Amelx*‑F | GTTGGGTTGGAGTCATGGAG | 162bp |  |
|  | *Amelx*‑R | GGCTGCACCACCAAATCATC |  |  |
| *Myog^1^* | *Om1a*^2^ | TTACGTCCATCGTGGACAGCAT | 246bp |  |
|  | *Om1b*^2^ | TGGGCTGGGTGTTAGTCTTAT |  |  |
|  |  |  |  |  |

^1^*Atr* is an alternative that is claimed to be superior for normalization (see [74]). ^2^See [110])

The genomic DNA can be prepared from as little tissue as an ear biopsy. We recommend DNA extraction to be followed by a purification step using, for instance, a phenol/chloroform protocol. The qPCR is performed according to the manufacturer’s instructions (see for example qPCR reagents SensiMix SYBR Lo-Rox as used by [73], or *Power* SYBR Green PCR Master Mix [74]). All reactions should be carried out in triplicate per assay and *Myog* included on every plate for normalization. We recommend using XY (1NPX) as the reference control, and known XY* (1NPX, 1NPX+), and XX (2NPX) samples in order to monitor the quality of your qPCR. A qPCR from a study that required XO and XY*^X^ mice [73] is shown in Additional Figure 2. A more relevant recent study provides a detailed protocol that was designed to identify a wider range of genotypes including XXY*^X^ [74]. This study utilized *Atr* on chromosome 9 for normalization rather than *Myog*.

The formula used to analyze raw data is a classic 2^(-ΔΔCT) [111]. Briefly, for each sample, the mean Ct for *Myog* is subtracted from the mean Ct for *Prdx4* or *Amelx* (ΔCT). Then the ΔCT of the reference DNA is subtracted from the ΔCT of samples (ΔΔCT). The formula 2^(- ΔΔCT) will then give an approximate copy number between 1 and 2. The copy number of the reference DNA will arbitrarily be set at 1. If the reference DNA is XX, we recommend multiplying the 2^(- ΔΔCT) by 2 in order to help data interpretation. Based on previous experience, data between 0.6 and 1.2 are considered 1 copy, and data between 1.6 and 2.2 are considered 2. To better distinguish between XY*^X^ and XXY*^X^ it would be possible to use fluorescent *in situ* hybridization (FISH) to count number of X chromosomes in interphase lymphocytes using the kit Kreatech KI-30505 RAB9B (XqF1). An alternative strategy would be to check for the *Xist* expression associated with two Xs (see [72]). The NPX qPCR can be utilized for all the progeny of the crosses listed in Additional file 1**.** The predicted NPX dosages are tabulated below including those for the recommended XY, XY* and XX controls.

| Genotypes | Gonadal sex | *Prdx4* copies  (NPX) | *Amelx* copies  (NPX and NPX+) | *Myog* copies  (Chromosome 1) |
| --- | --- | --- | --- | --- |
| Controls |  |  |  |  |
| 1,1,2 - XY | M | 1 | 1 | 2 |
| 1,2,2 - XY* | M | 1 | 2 | 2 |
| 2,2,2 - XX | F | 2 | 2 | 2 |
| **A**: XX x XY* |  |  |  |  |
| [XO rare] | F | 1 | 1 | 2 |
| XY*^X^ | F | 1 | 2 | 2 |
| XX | F | 2 | 2 | 2 |
| XY* | M | 1 | 2 | 2 |
| XX^Y*^ | M | 2 | 2 | 2 |
| **C**: XY*^X^ x XY |  |  |  |  |
| [XO rare] | F | 1 | 1 | 2 |
| XY*^X^ | F | 1 | 2 | 2 |
| XX | F | 2 | 2 | 2 |
| XXY*^X^ | F | 2 | 3 | 2 |
| **D**: XXY*^X^ x XY^-^*Sry* |  |  |  |  |
| XY^-^*Sry* | M | 1 | 1 | 2 |
| XX*Sry* | M | 2 | 2 | 2 |
| XXY*^X^*Sry* | M | 2 | 3 | 2 |
| XY^-^Y*^X^*Sry* | M | 1 | 2 | 2 |
| **B**: XY^-^Y*^X^*Sry* x XX |  |  |  |  |
| [XO rare] | M or F | 1 | 1 | 2 |
| XY^-^ | M or F | 1 | 1 | 2 |
| XY*^X^ | M or F | 1 | 2 | 2 |
| XX | M or F | 2 | 2 | 2 |
| XXY*^X^ | M of F | 2 | 3 | 2 |
| XY^-^Y*^X^ | M or F | 1 | 2 | 2 |
| XXY^-^ | M or F | 2 | 2 | 2 |
