# Supplementary material for: A primer on the use of mouse models for identifying direct sex chromosome effects that cause sex differences in non-gonadal tissues
Source: Biol Sex Differ. 2016 Dec 13;7:68. doi: 10.1186/s13293-016-0115-5 (PMC5154145; doi:10.1186/s13293-016-0115-5)
Supplement: Additional file 3: — Previously utilized MF1 crosses relevant to this review [10, 38, 67, 70, 71, 75, 77–79, 85, 112–115]. (DOCX 31 kb) [file 13293_2016_115_MOESM3_ESM.docx]

**Additional file 3. Previously utilized MF1 crosses relevant to this review**

*Alternative crosses* ***E*** *and* ***F*** *for detecting effects of parental X chromosome imprinting*

Female progeny from these crosses have previously been used to identify an effect of parental X imprinting on reversal learning of mice, which is a model of some behavioral differences found between X^p^O and X^m^O humans [85].

Cross **E:** XX x X*^Paf^*Y*.

Y* is the variant Y chromosome described for cross **A** [70, 71]. *Paf* (patchy fur) is a mutation linked to an inversion around the X-PAR boundary[112, 113]. The *Paf* mutation and Y* each involve chromosome rearrangements that lead to the generation of some sperm lacking a sex chromosome, and thus to XO offspring[112].

| Progeny^1^ | Gonads | Comments |
| --- | --- | --- |
| X^m^O | F | Approximately 40% of the female offspring are XO with a maternal X. |
| X^m^Y*^X^ | F |  |
| X^m^X^p,^*^Paf^* | F |  |

^1^ X^m^ maternal; X^p^ paternal, only female progeny are listed.

Cross **F:** In(X)*^Paf^*X x XY.

The mother carries one normal X together with an X chromosome with a large inversion [In(X)1H, abbreviated as In(X)]. Crossing over between the X and In(X) is associated with the frequent production of XO daughters - X^p^O and X^m^X^p^ are produced in approximately equal numbers, whereas X^m,^*^Paf^*X^p^ is produced less frequently [85, 114]. Female progeny carrying the In(X) were identified by an inversion-specific PCR and culled.

| Progeny^1^ | Gonads | Comments |
| --- | --- | --- |
| X^p^O | F | To check for an X imprinting effect the X^p^O females are compared with X^m^O females from cross **E**.  A check for confounding maternal effects is made by comparing *Paf*-carrying X^m^X^p^ females from the two crosses |
| X^m^X^p^ | F |  |
| X^m,^*^Paf^*X^p^ | F |  |
| [In(X)^m^X^p^] | F |  |
| [In(X)^m,^*^Paf^*X^p^] | F |  |

^1^ X^m^ maternal; X^p^ paternal, only female progeny are listed.

Cross **G:** XO x XY

In one study [67] cross **G** was used to generate X^p^O and X^m^X^p^ females to measure the effect of 1 *vs.* 2 X chromosomes [i.e. 1 *vs.* 2 (NPX + PAR)]. However, it is preferable for this comparison that the XO should be X^m^O because a single paternal X has a deleterious effect in early pregnancy [10, 75, 77-79]. The comparison of X^p^O *vs.* X^m^Y is also problematic as a test of the effect of adding a Y chromosome (NPY plus PAR), because of the conflicting X imprints, and because the presence of the Y chromosome is confounded with the effects of testicular hormones. The XO mothers are subfertile.

| Genotypes | Gonads | Comments |
| --- | --- | --- |
| X^p^O | F | This cross has been superseded by XX x XY* cross A, which is possible on MF1 and B6 backgrounds and avoids X^p^-only effects |
| X^m^X^p^ | F |  |
| X^m^Y | M |  |

Cross **H**: XX x X^Y*^O

Wijchers et al [38], when investigating the genetic basis of an increased frequency of silencing of a heterochromatin-sensitive transgene in males as compared to females, utilized the FCG cross to show that this XX *vs.* XY difference was independent of gonadal sex. They then used cross **H** to compare X^m^O *vs.* X^m^X^pY*^ to test whether this hormone independent direct SCE was linked to 1 vs 2 X chromosomes or to the absence *vs.* presence of a Y. The greater frequency of transgene silencing originally seen in XY males (compared to XX) was now seen in XO females, whereas the lower frequency previously seen in XX was found in XX^Y*^ males. This result confirmed the gonadal sex independence and showed that it correlated with the presence of a single (maternally derived) X. [NB. X^Y*^O males are typically sterile – fertile males on a predominantly MF1 background have been generated in the Burgoyne Lab [115]].

| Genotypes | Gonads | Comments |
| --- | --- | --- |
| X^m^O | F | Fertile X^Y*^O males are not available on a B6 background |
| X**^m^**X^pY*^ | M |  |

|  |  |  |
| --- | --- | --- |
|  |  |  |
