# Supplementary material for: A primer on the use of mouse models for identifying direct sex chromosome effects that cause sex differences in non-gonadal tissues
Source: Biol Sex Differ. 2016 Dec 13;7:68. doi: 10.1186/s13293-016-0115-5 (PMC5154145; doi:10.1186/s13293-016-0115-5)
Supplement: Additional file 4: — Crosses with Y chromosome translocations or deletions. (See chromosome diagrams in [55, 104, 116, 117]). (DOCX 20 kb) [file 13293_2016_115_MOESM4_ESM.docx]

**Additional file 4: Crosses with Y chromosome translocations or deletions** (See chromosome diagrams in [104])

The nine Y genes that have related X partner genes (Table 8) are prime candidates for NPY- dependent direct SCEs. All 9 genes are represented in a Yp derivative, *Sxr^a^* (officially designated as Tp(Y)1Ct*^Sxr-a^*), although the ~30 copies of *Rbmy* are reduced to an estimated 4 copies. *Sxr^a^* includes the testis determinant *Sry*, and when translocated to the distal tip of the X PAR generates XX*Sxr^a^* gonadal males. This sex reversing translocation is available on a B6 strain background (Jackson Labs strain 000552, B6.Cg-A^w-J^ Eda^Ta-6J^ Sxr). This stock is also carrying the X-linked marker Eda^Ta-6J^. This marker is redundant now that sex chromosome complement and the presence of *Sxr^a^* are easily monitored using PCR assays; Eda^Ta-6J^ is a deleterious mutation and can be eliminated by crossing to Jackson Laboratory B6 stock 000664.

Cross **I:** XX x XY*Sxr^a^*

In this cross *Sxr^a^* is initially located at the tip of the Y PAR. Due to PAR crossing over *Sxr^a^* is passed to half of the XX and XY progeny. Importantly, the XX*Sxr^a^* male progeny have no NPY genes from the long arm. Comparison of the results with those previously obtained from the FCG cross (section 4) should establish whether or not the direct SCE is due to a Yp-located NPY gene (or genes) within *Sxr^a^*.

| Genotypes | Gonads | Comments |
| --- | --- | --- |
| XX | F | XX*Sxr^a^* males are compared with XX and XY siblings to see if they exhibit the NPY-dependent SCE present in the XY males. If the *Sxr^a^* addition to XY increases the direct SCE, then this further supports Y short arm involvement. |
| XX*Sxr^a^* | M |  |
| XY | M |  |
| XY*Sxr^a^* | M |  |

Cross **J:** XX x XY^d1^*Sry*

If cross **I** above indicates that the NPY-dependent direct SCE is not due to NPY genes located in *Sxr^a^*, then it is still possible *Rbmy* is involved but the reduction from ~30 to 4 copies has rendered it ineffective. Alternatively, the direct SCE may depend on multi copy genes on the Y long arm (Yq). A Yp deletion variant, Y^d1^ that has only 1 or 2 copies of the *Rbmy*, but has a complete Y long arm, enables these alternatives to be resolved. A complication is that this deletion inhibits *Sry* transcription so that they develop as females[55, 116, 117]. The maintenance stock for the Y^d1^ deletion therefore utilizes the same *Sry* transgene as the FCG cross to compensate for the reduction in *Sry* expression. Those wishing to use this stock should contact [Andrew.Ojarikre@crick.ac.uk](mailto:Andrew.Ojarikre@crick.ac.uk). As with the FCG cross, the *Sry* transgene is located on chromosome 3 and segregates independently of the X and Y^d1^. Aside from the reduction in *Rbmy* copies in the Y bearing offspring the progeny are equivalent to those from the FCG cross (Table 2).

| Genotypes | Gonads | Comments |
| --- | --- | --- |
| XX | F | If the direct SCE was not manifest in *Sxr^a^* carriers, then one of two outcomes are expected: 1. The SCE is not manifest in XY^d1^ or XY^d1^,*Sry*; this implies that the SCE is *Rbmy* dependent. 2. If the SCE is manifest in these Y^d1^ carriers it implies the SCE is Yq gene dependent. |
| XX*Sry* | M |  |
| XY^d1^ | F |  |
| XY^d1^*Sry* | M |  |
|  |  |  |
